# Assessing the joint effects of education, economic status, empowerment, and employment (4Es) disparities on the co-coverage of maternal, newborn and child health care services in sub-Saharan Africa: an application of the intersectionality approach

**DOI:** 10.7189/jogh.15.04124

**Published:** 2025-05-09

**Authors:** Fati Kirakoya-Samadoulougou, Lucresse Corine Fassinou, Mahaman Lawali Inoussa Garba, Abdoulaye Maïga, Scott L Zeger, Agbessi Amouzou

**Affiliations:** 1 Department of International Health, Johns Hopkins Bloomberg School of Public Health, Baltimore, Maryland, USA; 2 Centre de Recherche en Épidémiologie, Biostatistique et Recherche Clinique, École de santé publique, Université libre de Bruxelles, Brussels, Belgium; 3 Department of Biostatistics, Johns Hopkins Bloomberg School of Public Health, Baltimore, Maryland, USA

## Abstract

**Background:**

Although education, employment, economic status, and empowerment (4Es) are known to individually influence inequalities in maternal, newborn, and child health (MNCH), their combined effects have not been thoroughly studied in sub-Saharan Africa (SSA). We applied an intersectional approach to understand the joint effect of the 4Es on MNCH co-coverage in different settings in SSA.

**Methods:**

We used 25 Demographic and Health Survey data sets and employed a multilevel analysis of individual heterogeneity and discriminatory accuracy to assess the intersectional effects of the 4Es on MNCH service co-coverage and inequalities within and across countries. The variance partition coefficient and proportional change in variance (PCV) statistics were applied to quantify total intersectional effects.

**Results:**

Among 103 388 women with children aged 12–59 months, 4.6% of the variance in co-coverage of ≥6 MNCH interventions (co-coverage ≥6) occurred at the intersectional strata level. Most of this variance (90.3%) was due to the additive effects of the 4Es, with education (PCV partial = 80.7%) the primary contributor, followed by economic status (PCV partial = 9.8%). The lowest co-coverage was observed among women with no education, unemployment, low economic status, and low empowerment. Inequalities were more pronounced in countries with lower universal health coverage (UHC) indices, where co-coverage ranged from 17.5% (95% confidence interval (CI) = 14.6–21.1) to 67.0% (95% CI = 62.9–70.8), compared with 42.8% (95% CI = 38.0–47.8) to 68.5% (95% CI = 64.7–71.8) in countries with higher UHC indices. Evidence of multiplicative effects was also observed. Services with a high disparity included skilled birth attendance, antenatal care, and access to improved water sources. Country-specific analysis revealed that 11 countries showed very low heterogeneity (<5%) in the co-coverage of ≥6 interventions.

**Conclusions:**

This is the first study to explore how the 4Es jointly affect MNCH co-coverage in SSA. The results reveal that these 4Es are connected and affect MNCH co-coverage, particularly in key services, including skilled birth attendance, antenatal care, and access to improved water sources. The most privileged groups had significant protective effects, whereas those with fewer societal privileges showed minor effects. Learning from countries with low disparities in service co-coverage can help reduce the gaps in other countries.

In September 2015, the United Nations General Assembly replaced the Millennium Development Goals with the Sustainable Development Goals (SDGs) [1], a universal call to action aimed at eradicating poverty, protecting the environment, and promoting global well-being by 2030 [2,3]. Among the 17 SGDs, SDG-3 focusses on ‘ensuring healthy lives and promoting well-being for all at all ages’ [4]. Despite various scientific, technical, and political strategies implemented by the global community to achieve health-related goals, the world remains significantly off-track, particularly concerning maternal and child mortality [5,6]. Maternal mortality, which significantly declined during the Millennium Development Goals, stagnated between 2016 and 2020, with 287 000 maternal deaths (800 per day) in 2020. Notably, 70% of these deaths were concentrated in low- and middle-income countries, specifically in sub-Saharan Africa (SSA) [7]. Similarly, challenges persist in reducing under-five mortality and neonatal mortality rates in SSA. Most deaths are preventable through effective interventions such as skilled birth attendance, emergency obstetric care, vaccinations, nutritional supplementation, and water and sanitation programmes [8,9]. The COVID-19 pandemic has exacerbated these disparities, particularly among the most vulnerable populations, highlighting the inadequacy of isolated approaches to health [6,8,10].

Maternal, newborn, and child health (MNCH) interventions are implemented concurrently in most low- and middle-income countries [11,12]. Increased coverage of these interventions is projected to avert 71% of neonatal deaths, 33% of stillbirths, and 54% of maternal per year [13]. Achieving this goal requires equal access for all women and their children to provide comprehensive and high-quality MNCH services. However, in SSA, inequalities in MNCH service coverage hinder progress towards SDG-3 [14]. To assess progress and identify underserved populations, the Child Health Epidemiology Reference Group has developed tools to track MNCH intervention coverage [15]. One such tool is co-coverage measurement, which counts the number of preventive interventions received by individual mother-child pairs out of a set of eight interventions: at least one antenatal care (ANC) visit; tetanus vaccination during pregnancy; skilled birth attendance (SBA); vaccination against tuberculosis, diphtheria, pertussis, tetanus, and measles; childhood Vitamin A supplementation; and access to improved drinking water in the household [16].

Numerous studies have demonstrated a positive relationship between MNCH interventions and factors such as women’s education, empowerment, employment, and socioeconomic background, collectively referred to as the 4Es [17–25]. The PROGRESS framework also considers these four social dimensions, which can be used to assess the effects of interventions on health equity [26]. These four dimensions align with several SDGs, including SDG1 (*i.e.* poverty eradication), SDG4 (*i.e.* quality education), SDG5 (*i.e.* gender equality), and SDG8 (*i.e.* decent work and economic growth) and serve as key structural determinants of health care access and use. Female education has been proven to be a strong predictor of the use of maternal health care services [18,27]. It sustains healthy lifestyles and positive choices by promoting health awareness and the likelihood of seeking high-quality health care services [28]. It has also been proven to enhance women’s empowerment by improving the view of women on their health [27], employment by offering more opportunities for work [29], and economic status by favouring a better standard of life [18]. All these factors align directly with specific SDGs, including poverty eradication, quality education, gender equality, decent work and economic growth, and reduced inequalities [3,18]. To achieve SDGs by 2030 in SSA, it is necessary to understand how these factors contribute to improvements in MNCH indicators.

In the urban settings of SSA, unequal utilisation of MNCH services has been linked to low levels of education, unemployment, lower socioeconomic status, and poor living conditions [30]. An evaluation of equity in maternal health services across 25 SSA countries revealed that women with higher education levels, greater exposure to mass media (*i.e.* radio and TV), and higher household wealth were more likely to complete the continuum of care [31]. Despite the well-established individual associations of the 4Es with MNCH coverage, essential questions about their combined effects remain unanswered. Women often face multiple intersecting dimensions of inequity that can create complex patterns of inequity between socially disadvantaged groups and those with more advantaged identities [32]. Unidimensional analyses are insufficient for understanding the patterns of inequality in MNCH coverage among women with multiple disadvantages.

To address this issue, the intersectionality approach has been increasingly used in equity research [33–35]. The approach assumes that multiple social identities intersect at the micro level of individual experiences to reflect social positions, such as privilege and oppression, operating at the macro socio-structural level [36]. This framework is based on Black feminist theory, which recognises that an individual's position at the crossroads of multiple social identities (*e.g.* gender, race, and class) leads to distinct lived experiences that affect health [37]. Multilevel analysis of individual heterogeneity and discriminatory accuracy analysis is the most used quantitative modelling which captures intersectional inequities. This is due to its multilevel structure, where individuals are nested within social strata that are defined by combinations of social position variables, and because of its capacity to differentiate additive vs multiplicative (interactive) intersectional effects [38]. The additive effects consider the effects of social identities singly and assume that effects at an intersection of identities can be understood as a sum of their parts. Multiplicative intersectional effects, in contrast, assume that experiences at an intersection are co-constituted and must be considered jointly [39,40].

In the context of health equity research in SSA, the intersectionality framework is especially pertinent due to the region's profound social, economic, and cultural diversity, which often contributes to complex health disparities. Women in SSA frequently face multiple, overlapping disadvantages, including lower educational attainment, economic hardship, unemployment, limited empowerment, and systemic barriers within the health care system [41]. However, to our knowledge, no study has examined how different socioeconomic markers interact to create inequalities in MNCH co-coverage in SSA. We address this gap by exploring intersectional inequities in MNCH interventions, specifically examining how the joint effects of the 4Es factors influence MNCH co-coverage in SSA.

## METHODS

### Data source and study population

We used standard Demographic and Health Survey (DHS) data from 25 countries in SSA (Table S1 in the **Online Supplementary Document**). These countries were selected based on the availability of standard DHS conducted between 2015–22. This time frame aligns with the post-2015 era following the launch of the SDGs, which set new targets for improving health outcomes, including reducing maternal and child mortality. The earliest DHS data were from Angola (2015/2016), and the most recent were from Ghana, Kenya, and Tanzania (2022). Each DHS survey was cross-sectional and nationally representative, using probability-based multistage clustered sampling of households.

Within each sampled household, women aged 15–49 were asked to provide information about themselves, their households, and their children. For this study, we used individual women’s data sets (individual recode) and included women aged 15–49 who reported having at least one live birth within the five years preceding the survey. To mitigate recall bias and minimise missing information, we focussed on the most recent live births reported by each participant if they had multiple births within the five-year recall period. We only included women in couples in the analysis because the denominator for calculating women’s empowerment was based on married women.

### Dimensions of social strata

We created the intersectional strata based on the dimensions of each social factor. We constructed two strata levels: the 4Es level (considering only the 4Es) and the 4Es and universal health coverage (UHC) level (considering the 4Es and the national UHC context). There were three categories for education, two for employment, five for economic status, three for empowerment, and two for UHC, resulting in 90 strata for the 4Es-level (3 × 2 × 5 × 3) and 180 strata for the 4Es and UHC level (3 × 2 × 5 × 3 × 2).

To construct the social strata, we retained the existing coding, as found in the DHS, for level of education, wealth quintile, and current employment status (Table S2 in the **Online Supplementary Document**).

Regarding the variable related to women’s empowerment, the capacity to participate in all decision-making was defined as a woman making all three specific decisions (*i.e.* own health care, large household purchases, and visits to family or relatives) either alone or jointly with her husband. The capacity to disagree with all the specific reasons justifying wife-beating was related to burning food, arguing with the husband, going out without telling him, neglecting children, and refusing sexual intercourse with him. Women who participated in all decision-making processes and disagreed with the specific reasons justifying wife-beating were considered to have high empowerment levels. When only one of the conditions was fulfilled, the women were said to have a middle empowerment level. When they could not decide and/or disagreed with any reason for wife-beating, they were classified as having a low empowerment level. This definition is based on a calculation proposed by the DHS [42]. The variable related to employment was based on the question: ‘Have you done any work in the last 12 months?’ [43]. This definition included formal and informal work and was used in binary form (Table S2 in the **Online Supplementary Document**).

To account for a country's contextual situation, we considered the UHC index, defined according to the World Health Organization (WHO), as the capacity of the country to offer access to the full range of quality health services people need, when and where they need them, without financial hardship. For both indices, the median was calculated, and each country with at least one median value was considered to have a high UHC index. Otherwise, it was classified as a low-UHC country.

### Outcome

The outcome of this study was the co-coverage of the MNCH interventions, which was assessed using the co-coverage index. The index is calculated at the mother-child pair level and is defined as the total number of interventions received from the following eight: at least one ANC visit with a skilled provider (Table S3 in the **Online Supplementary Document**); tetanus vaccination during pregnancy; skilled birth attendant (SBA); child received vitamin A supplementation; *Bacillus Calmette-Guerin* (BCG) vaccination; DPT3 vaccination (*i.e.* a combination of vaccines against diphtheria-tetanus-pertussis); measles vaccinations; and access to an improved drinking water source in the household.

Following the WHO recommendations, tetanus vaccination was defined as receiving at least two tetanus injections during the most recent pregnancy. Women whose more recent births were protected against neonatal tetanus were classified as having received tetanus vaccination. The co-coverage index is a globally validated indicator used to track progress in health care access [44,45]. The index is the percentage of the mother-child pairs covered by ≥3 or ≥6 interventions. Each mother-child pair received a score of 0–8, with all indicators calculated for children aged 12–59 months. Children <12 months of age were excluded because some interventions, such as measles vaccination, are typically administered between 9 and 12 months in most countries [12,46]. For the multilevel analysis of individual heterogeneity and discriminatory accuracy (MAIHDA) analysis, the co-coverage was redefined into a binary variable with a cutoff of 6 interventions (0 if <6 interventions and 1 if ≥6 interventions).

### Covariates

Consistent with previous studies, we controlled for women’s characteristics that were identified a priori as being associated with the uptake of MNCH services [47–49]. These covariates included the woman’s age (15–24, 25–34, and 35–49), parity (1, 2–4, and ≥5), place of residence (rural or urban), and access to mass media (yes or no), which was defined as exposure to specific media (newspaper, TV, or radio) weekly. We also included the country as a covariate to consider the specific differences between countries.

### Statistical methods

We conducted a descriptive analysis of maternal characteristics, and numbers and percentages were reported for categorical variables, whereas means and 95% confidence intervals (CI) were presented for quantitative variables. Data were weighted for descriptive analysis to account for the complex survey design of the DHS, ensuring that the results were representative of the population and that the estimates accurately reflected the distribution of MNCH service coverage across different social strata within each country.

To examine intersectionality, we conducted MAIHDA [33,50]. This analysis assessed individuals nested within social strata, allowing a detailed evaluation of how factors at different levels contribute to variations in the continuum of care. We considered the 4Es level and 4Es combined with country-level characteristics (UHC index) in the analysis.

Following the MAIHDA methodology [33,50], we used multilevel logistic regression models to estimate the effects of social strata on co-coverage. In these models, women represent the first level (level 1) and are clustered within the social strata (level 2). The MAIHDA models were applied in two contexts: 4Es intersectional strata and 4Es combined with the national context (UHC) strata. Three models were run for the analysis. Model 1, the simple intersectional model, was an empty random-intercept model estimating the variance partitioning coefficient (VPC). In the context of 4ES intersectional strata, model 2 included five sub-models (2a–e). Models 2a, 2b, 2c, and 2d (partially adjusted intersectional models) were individually adjusted for each main effect (4Es), and model 2e (the intersectional interaction model) included all the main effect variables simultaneously. In the context of 4Es combined with the national context (UHC) strata, model 2 included six sub-models (2a–f). Models 2a, 2b, 2c, 2d and 2e (partially adjusted intersectional models) were individually adjusted for each main effect (4E and UHC), and model 2f (the intersectional interaction model) included all the main effect variables simultaneously. Model 3 consisted of adding covariates to models 2e (4Es-social strata) and 2f (4Es combined with the national context (UHC) social strata). Additionally, sensitivity analyses were done to evaluate the robustness of the findings. These analyses included adjusting model 2e or 2f (depending on the context), as well as model 3 for the country variable.

We presented the results from these models as odds ratios (OR) with 95% CI and proportional change in variance (PCV). The PCV quantifies the extent to which the social dimensions individually and jointly contribute to the clustering estimated in model 1. In other words, PCV estimates the variation between strata attributable to the main effects, while 1-PCV measures the proportion of the variation due to the interaction of effects or the effect of other variables not included in the model. This analysis shows how much interaction effects are necessary to explain the disparities between strata (**Online Supplementary Document**).

The variables defining these contexts were included in level 2, with individuals nested within these intersectional strata at level 1. While the primary objective of our study was to examine the overall effect of intersectionality across SSA, we acknowledge the importance of considering within-country heterogeneity due to each country's specific cultural realities. We conducted additional country-specific analyses (Tables S7 and S8 in the **Online Supplementary Document**). These analyses allow for a more nuanced understanding of national-level disparities.

### Software

All models were fitted to the *R*, version 4.3.1 (R Core Team, Vienna, Austria) using the lme4 package [51].

## RESULTS

**Figure 1:**
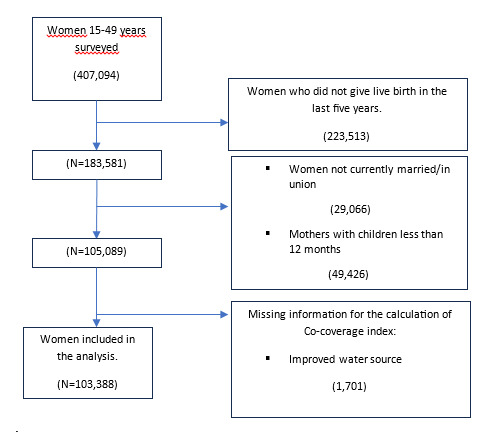
Flowchart of women included in the study.

A total of 103 388 women in couples with living children aged 12–59 months were included in the analysis (**Figure 1**). Sociodemographic analysis revealed that most women in the study were aged 25–34 years (48.3%), had given birth 2–4 times (51.0%), lived in rural areas (66.0%), and did not have access to mass media (52.6%). More than half of the participants were currently employed (71.1%) and had a moderate level of empowerment (56.1%). On average, each woman-child pair received five interventions, with 89% receiving ≥3 interventions and approximately half receiving ≥6 interventions. A total of 2429 women (2.3%; 95% CI = 2.1–2.6) did not complete any interventions, while 17.9% (95% CI = 17.5–18.3) completed all eight interventions.

The social strata most likely to receive ≥6 interventions included: women currently employed (52.4%), with a secondary or higher education level (61.7%), belonging to the richest wealth quintile (58.6%), and having a middle level of empowerment (52.3%) (**Table 1**). Analysis of the individual components of co-coverage revealed that antenatal care with a skilled provider was the most utilised intervention (87.2%), followed by access to improved water sources (74.2%), SBA (73.2%), and tetanus vaccination during pregnancy (70.3%) (Table S4 in the **Online Supplementary Document**).

**Table 1:** Baseline characteristics of women who gave live birth in the last five years in sub-Saharan Africa, 2015–22 (n = 103 178)

	n (%)	Number of interventions, x̄ (SE)	Percentage of participants who had ≥6 interventions, x̄ (95% CI)
**Total**	103 178 (100.0)	5.4 (0.02)	51.7 (51.0–52.3)
**Currently employed**			
No	29 808 (28.9)	5.2 (0.03)	49.7 (48.7–50.8)
Yes	73 370 (71.1)	5.4 (0.02)	52.4 (51.8–53.1)
**Education level**			
None	38 294 (37.1)	4.6 (0.03)	39.9 (38.9–40.9)
Primary	33 934 (32.9)	5.6 (0.02)	55.8 (55.0–56.6)
Secondary/higher	30 950 (30.0)	6.0 (0.01)	61.7 (60.9–62.5)
**Economic status**			
Poorest	21 390 (20.7)	4.8 (0.03)	43.9 (42.8–45.0)
Poorer	21 076 (20.4)	5.2 (0.03)	49.1 (48.0–50.1)
Middle	21 140 (20.5)	5.4 (0.03)	52.0 (54.5–56.6)
Richer	20 633 (20.0)	5.6 (0.02)	55.6 (54.5–56.6)
Richest	18 939 (18.4)	5.9 (0.02)	58.6 (57.7–59.6)
**Women empowerment**			
Low	27 979 (27.1)	5.3 (0.03)	51.7 (50.7–52.7)
Middle	57 920 (56.1)	5.4 (0.02)	52.3 (51.6–53.0)
High	17 279 (16.8)	5.3 (0.03)	49.5 (48.4–50.7)
**Age in years**			
15–24	23 741 (23.0)	5.5 (0.02)	56.8 (55.8–57.8)
25–34	49 872 (48.3)	5.5 (0.02)	53.9 (53.1–54.6)
35–49	29 565 (28.7)	5.1 (0.02)	43.8 (43.0–44.6)
**Parity**			
1	17 472 (16.9)	5.7 (0.02)	58.8 (57.8–59.9)
2–4	52 622 (51.0)	5.5 (0.02)	54.3 (53.6–55.0)
≥5	33 084 (32.1)	4.9 (0.02)	43.7 (42.8–44.6)
**Area of residence**			
Urban	35 108 (34.0)	5.8 (0.02)	57.6 (56.7–58.5)
Rural	68 070 (66.0)	5.1 (0.02)	48.6 (47.8–49.4)
**Mass media access**			
No	54 238 (52.6)	4.9 (0.02)	45.0 (44.2–45.8)
Yes	48 940 (47.4)	5.8 (0.01)	59.1 (58.4–59.7)
**Belonging to a country with a high UHC index**			
No	67 293 (65.2)	5.1 (0.02)	46.4 (45.6–47.2)
Yes	35 885 (34.8)	5.9 (0.02)	61.6 (60.8–62.4)

CI – confidence interval, SE – standard error, UHC – universal health coverage, x̄ – mean

Ghana had the highest proportion of mother-child pairs with a co-coverage of ≥6 interventions (89.3%), while Ethiopia had the lowest proportion (23.9%) (Figure S1 in the **Online Supplementary Document**).

Considering the 4Es social strata for the models, 90 strata were created, with sample sizes of 101–5000. When considering the 4Es and country-level social strata, 180 were formed, with sample sizes of 101–10 000 (Table S5 in the **Online Supplementary Document**).

### 4Es main effects

The inclusion of the 4Es individually in the models showed that women with primary (OR = 1.88; 95% CI = 1.71–2.07) or secondary/higher education level (OR = 2.33; 95% CI = 2.11–2.56), and those belonging to the richer (OR = 1.34; 95% CI = 1.04–1.72) and richest wealth quintiles (OR = 1.40; 95% CI = 1.08–1.80) were significantly more likely to receive ≥6 interventions compared to those with no education and belonging to the poor or middle wealth quintiles. The status of employment also showed a significant association in model 2e, adjusted for the 4Es (OR = 0.97; 95% CI = 0.94–0.99), and model 3, adjusted for both the 4Es and covariates (OR = 0.96; 95% CI = 0.94–0.99).

The VPC of the empty model (model 1) was 4.6%, indicating that approximately 5% of the total variance in the co-coverage of ≥6 interventions was explained by the intersectional strata level. This means that approximately 5% of the difference in receiving ≥6 interventions can be attributed to the specific combination of education, employment, economic status, and empowerment levels experienced by each woman. This variance was substantially reduced to 0.5% when the dimensions of social strata were incorporated as additive main effects (model 2e). The inclusion of these main effects accounted for 90.3% of the between-strata variance (*i.e.* PCV), indicating that about 10% of the total variance was not explained by the addition of the main effects, which could be attributable to intersectional effects or other variables not included in the models. After adjusting for covariates, VPC remained unchanged. When analysing each component of co-coverage, VPC was highest for SBA (15.3%), followed by ANC with a skilled provider (14.5%) and access to an improved water source (10.8%). The other components showed low heterogeneity between the strata, with VPC values ranging from 2.0% for BCG vaccination to 4.8% for tetanus vaccination (Table S6 in the **Online Supplementary Document**).

At the country level, high VPC values (>10%) for co-coverage of ≥6 were observed in eight countries: Kenya (26.0%), Nigeria (19.7%), Tanzania (19.7%), Angola (18.6%), Ghana (15.7%), Ethiopia (13.8%), Guinea (13.5%), and Burkina Faso (11.9%), suggesting significant heterogeneity within these countries (Table S7 in the **Online Supplementary Document**). Conversely, 11 countries exhibited very low heterogeneity (<5%): Burundi (0.4%), Zimbabwe (0.4%), Zambia (0.5%), Sierra Leone (0.7%), Malawi (0.9%), South Africa (1.2%), Uganda (1.4%), Gambia (1.8%), Liberia (3.1%), Mauritania (4.5%), and Gabon (4.9%), suggesting more uniform access and use of ≥6 MNCH services between social strata. Of the countries with very low heterogeneity, three (*i.e.* Gabon, Liberia, and South Africa) exhibited universally low access to specific services such as DPT3 and MSL vaccination, BCG vaccination in South Africa, and vitamin A supplementation in Gabon. This indicates that while notable inequalities in access to MNCH services were not noticed within these countries, systemic challenges still exist in ensuring universal coverage of specific health interventions.

In countries with high heterogeneity in the co-coverage of ≥6 interventions, certain services exhibited exceptionally high levels of variability. ANC1, SBA, BCG, DPT3, MSL, and access to improved water sources showed significant disparities between strata in Kenya. In Tanzania, ANC1, SBA, and DPT3 vaccinations and access to improved water sources were the main contributors to heterogeneity. In Nigeria, ANC1, tetanus vaccination during pregnancy, SBA, vitamin A supplementation, BCG vaccination, DPT3 vaccination, and improved water sources were highly variable between the strata. Ghana presented significant disparities in ANC1, SBA, and access to improved water sources, whereas in Guinea, SBA and access to improved water sources were the primary drivers of heterogeneity. Angola exhibited notable variability in ANC1, SBA and access to improved water sources. In Burkina Faso, SBA and BCG vaccination were the services with the most heterogeneity between strata, whereas ANC1, vaccination against tetanus in pregnancy, SBA, and access to improved water sources exhibited significant variability in Ethiopia (Table S8 in the **Online Supplementary Document**).

The analysis of the partially adjusted intersectional models (2a, 2b, 2c, 2d) aimed at evaluating the contribution of each 4Es variable to inequalities revealed that women’s educational level was the most significant contributor, with a PCV of 80.7%, followed by economic status (PCV = 9.8%) (**Table 2**). By contrast, employment and women’s empowerment had low effects on inequalities, with PCVs of 0.8% and 0.6%, respectively. When stratified by women’s educational level, among those with no education, the highest predicted proportion of women with co-coverage of ≥6 interventions was 50.1% (95% CI = 46.0–54.1), and the lowest was 29.7% (95% CI = 26.8–32.7). In the social strata of women with primary education, the highest predicted proportion was 60.3% (95% CI = 56.4–64.2), and the lowest was 52.4% (95% CI = 47.8–56.7). For women with secondary or higher levels of education, the highest predicted proportion was 68.5% (95% CI = 64.7–71.8), and the lowest was 53.7% (95% CI = 48.1–59.0).

**Table 2:** Multilevel model estimates of co-coverage of ≥6 interventions among women and children in sub-Saharan Africa (2015–22), clustered by 4Es (n = 103 324)

	Model, OR (95% CI)						
	**1: empty model**	2a: education*	2b: employment*	2c: economic status*	2d: empowerment*	2e: main effects*	**3: adjusted for covariates†**
**Intercept**	1.11 (1.02–1.20)	0.68 (0.63–0.73)	1.13 (1.04–1.23)	0.92 (0.77–1.10)	1.13 (0.98–1.30)	0.59 (0.54–0.65)	0.95 (0.85–1.06)
**Educational level**							
None		ref				ref	ref
Primary		1.88 (1.71–2.07)				1.87 (1.73–2.01)	1.71 (1.59–1.84)
Secondary/higher		2.33 (2.11–2.56)				2.29 (2.12–2.46)	1.79 (1.66–1.93)
**Currently employed**							
No			ref			ref	ref
Yes			1.07 (0.91–1.26)			1.07 (1.01–1.14)	1.12 (1.05–1.19)
**Economic status**							
Poorest				ref		ref	ref
Poorer				1.12 (0.87–1.43)		1.12 (1.02–1.23)	1.09 (1.00–1.20)
Middle				1.21 (0.94–1.55)		1.20 (1.10–1.32)	1.17 (1.07–1.28)
Richer				1.34 (1.05–1.71)		1.33 (1.21–1.46)	1.30 (1.19–1.43)
Richest				1.40 (1.10–1.78)		1.38 (1.25–1.52)	1.35 (1.23–1.49)
**Women empowerment**							
Low					ref	ref	ref
Middle					1.01 (0.83–1.23)	1.01 (0.94–1.08)	1.02 (0.95–1.09)
High					0.94 (0.76–1.15)	0.94 (0.87–1.02)	0.98 (0.91–1.05)
**Random effects**							
Strata, n	103 388	103 388	103 388	103 388	103 388	103 388	103 388
VPC, %	4.6	0.9	4.6	4.2	4.5	0.5	0.5
PCV, %		80.7	0.8	9.8	0.6	90.3	90.7
AUC, %	61.2					61.2	

AUC – area under curve, CI – confidence interval, OR – odds ratio, PCV – proportional change in variance, ref – reference, VPC – variance partitioning coefficient, 4Es – education, employment, economic status, and empowerment

*Models 2a, 2b, 2c, and 2d were partially adjusted for each 4Es variable, and model 2e was adjusted for all 4Es variables.

†Model 3 was adjusted for age, parity, residence area, and mass media access.

**Figure 2:**
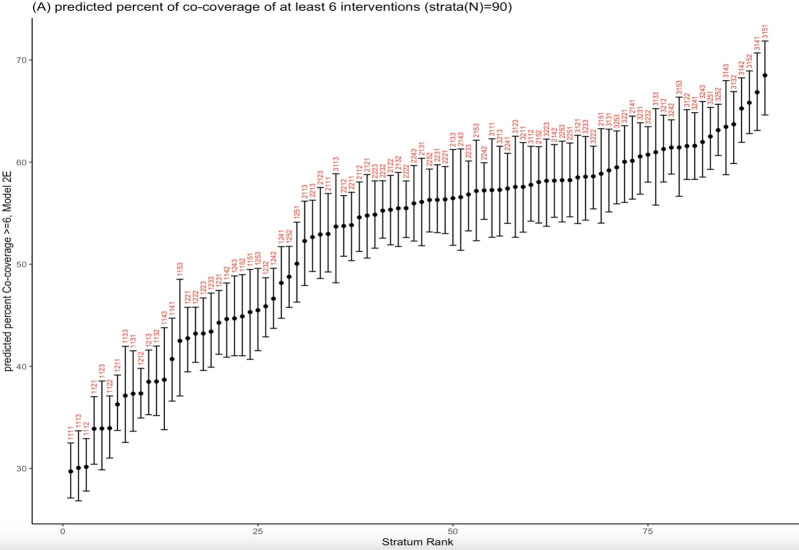
Predicted percent of co-coverage of ≥6 interventions. Strata (n = 90) are identified by a four-digit label corresponding to the four dimensions (4Es) in the following order: educational level: 1 = no education, 2 = primary, 3 = secondary/higher; employment: 1 = not currently employed, 2 = currently employed; economic status: 1 = poorest, 2 = poorer, 3 = middle, 4 = richer, 5 = richest; and empowerment: 1 = low, 2 = middle, 3 = high. CI – confidence interval.

The plot of the predicted strata-level proportions of women-child pairs participating in ≥6 interventions, with their 95% confidence intervals ranked from lowest to highest, shows that the lowest predicted proportion was found in stratum 1111, which comprised women with no education, no current employment, belonging to the poorest wealth quintile, and a low level of empowerment (**Figure 2**). The highest predicted proportion was observed in stratum 3151, which comprised women with secondary or higher levels of education, no current employment, belonging to the richest wealth quintile, and a low level of empowerment. The predicted proportions ranged from 29.7% (95% CI = 29.6–32.7) to 68.5% (95% CI = 64.7–71.8).

For the 4Es-strata level, the estimates of intersectional effects (random effects) for the co-coverage of ≥6 interventions for model 2 ranged from −0.27 to 0.21, with 18 out of 90 strata’s 95% CI not including zero (20.0%), indicating the presence of intersectional effects (multiplicative effect) (Figure S2 in the **Online Supplementary Document**).

### Country-level main effects

When the country’s UHC index was added to the models, the VPC increased slightly to 5.6% in the empty model (**Table 3**). After adding the main effect variables, VPC decreased to 1.0. The increase in VPC from the simple 4Es-strata to the 4Es and UHC strata suggests that differences in UHC levels between the countries may explain a moderate proportion of the variability in co-coverage with ≥6 interventions. When the models were adjusted for covariates, the VPC decreased slightly to 0.9. The area under the curve increased slightly to 63.1% in the simple 4Es-level models. The partially adjusted intersectional model analysis revealed that educational level (PCV = 44.8%), the country’s UHC index (PCV = 30.7%), and economic status (PCV = 9.9%) contributed substantially to inequalities (Table S9 in the **Online Supplementary Document**).

**Table 3:** Multilevel model estimates of co-coverage of ≥6 interventions among women and children in SSA (2015–22), clustered by 4Es and UHC index

	Model, OR (95% CI)							
	**1: empty model**	2a: education*	2b: employment*	2c: economic status*	2d: empowerment*	2e: country’s UHC index*	2f: main effects*	**3: adjusted for covariates†**
**Intercept**	1.19 (1.11–1.27)	0.80 (0.73–0.87)	1.07 (0.87–1.32)	0.95 (0.82–1.08)	1.21 (1.08–1.36)	0.93 (0.86–1.01)	0.47 (0.41–0.54)	0.75 (0.65–0.87)
**Educational level**							ref	ref
None		ref					1.64 (1.52–1.76)	1.50 (1.40–1.62)
Primary		1.65 (1.45–1.86)					1.94 (1.80–2.10)	1.53 (1.42–1.66)
Secondary/higher		1.99 (1.76–2.26)						
**Currently employed**								
No			ref				ref	ref
Yes			1.07 (0.94–1.23)				1.08 (1.01–1.15)	1.13 (1.07–1.20)
**Economic status**				ref				
Poorest				1.18 (0.97–1.44)			ref	ref
Poorer				1.28 (1.06–1.56)			1.17 (1.06–1.29)	1.15 (1.04–1.26)
Middle				1.43 (1.17–1.74)			1.26 (1.15–1.39)	1.24 (1.13–1.37)
Richer				1.48 (1.21–1.80)			1.41 (1.28–1.55)	1.40 (1.27–1.54)
Richest							1.45 (1.31–1.60)	1.45 (1.32–1.61)
**Women empowerment**								
Low					ref		ref	ref
Middle					0.99 (0.85–1.17)		0.99 (0.92–1.06)	1.00 (0.93–1.07)
High					0.90 (0.77–1.06)		0.90 (0.83–0.97)	0.93 (0.86–1.01)
**Country with high UHC index**								
No						ref	ref	ref
Yes						1.64 (1.46–1.83)	1.61 (1.52–1.72)	1.61 (1.52–1.72)
**Random effects**								
Strata, n	103 388	103 388	103 388	103 388	103 388	103 388	103 388	103 388
VPC, %	5.6	3.2	5.5	5.1	5.5	3.9	1.0	0.9
PCV, %		44.8	0.7	9.9	1.0	30.7	83.6	84.2
AUC, %	63.1						63.1	

AUC – area under curve, CI – confidence interval, SSA – sub-Saharan Africa, OR – odds ratio, PCV – proportional change in variance, ref – reference, UHC – universal health coverage, 4Es – education, employment, economic status, and empowerment

*Models 2a, 2b, 2c, 2d, 2e were partially adjusted for each individual 4Es variables, and model 2f was adjusted for all 4Es variables and the UHC variable.

†Model 3 was adjusted for age, parity, residence area, and mass media access.

**Figure 3:**
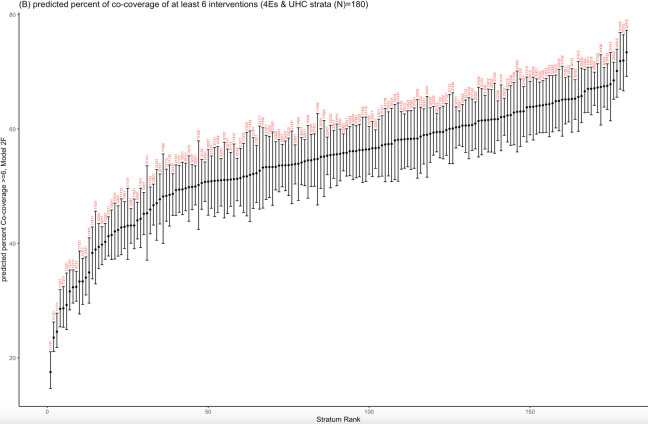
Predicted percent of co-coverage of ≥6 interventions. Strata (n = 180) are identified by a four-digit label corresponding to the four dimensions (4Es) in the following order: educational level: 1 = no education, 2 = primary, 3 = secondary/higher; employment: 1 = not currently employed, 2 = currently employed; economic status: 1 = poorest, 2 = poorer, 3 = middle, 4 = richer, 5 = richest; empowerment: 1 = low, 2 = middle, 3 = high; and country with high UHC index: 1 = no, 2 = yes. CI – confidence interval.

When considering the 4Es and UHC strata, the lowest predicted proportion of women-children pairs with co-coverage of ≥6 interventions (17.4%; 95% CI = 14.4–20.4) was observed in stratum 11131, comprising women with no level of education, no current employment, belonging to the poorest wealth quintile group, having a high level of empowerment, and belonging to a country with low UHC index (**Figure 3**; Table S10 in the **Online Supplementary Document**). The highest predicted proportion (73.6%; 95% CI = 69.3–77.0) was observed in stratum 32412, composed of women with secondary or high levels of education, current employment, belonging to the wealthier quintile, having a low level of empowerment, and living in a country with a high UHC index.

When stratified by the UHC index, among women living in a country with a low UHC index, the highest predicted proportion (67.0%; 95% CI = 62.9–70.8) was observed in stratum 31511, represented by women with secondary or higher educational level, no current employment, belonging to the richest wealth quintile, and with a low level of empowerment. When stratified by educational level in the same social stratum, in women with no education, the highest predicted proportion of co-coverage of ≥6 interventions was 49.4% (95% CI = 45.0–54.2), and the lowest was 17.5% (95% CI = 14.6–21.1). Among those with a primary education level, the highest predicted proportion was 56.7% (51.6–61.7), and the lowest was 43.1% (95% CI = 37.2–49.6). In women with secondary/higher education, the highest predicted proportion was 67.0% (95% CI = 62.9–70.8), and the lowest was 45.3% (95% CI = 37.1–53.6).

Among women living in a country with a high UHC index, the lowest predicted proportion (42.8%; 95% CI = 38.0–47.8) was observed in stratum 11112, composed of women with no educational level, unemployed, belonging to the poorest wealth quintile group, and with a low level of empowerment. These results suggest higher intersectional inequalities in countries with low levels of UHC. When stratified by educational level in the same social stratum, in women with no educational level, the highest predicted proportion of co-coverage of ≥6 interventions was 61.6% (95% CI = 56.0–67.0), and the lowest was 42.8% (95% CI = 38.0–47.8). In those with a primary educational level, the highest predicted proportion was 67.8% (95% CI = 61.4–73.3), and the lowest was 56.4% (95% CI = 50.8–62.1). In women with secondary/higher education, the highest predicted proportion was 73.4% (95% CI = 69.2–77.2), and the lowest was 57.3% (95% CI = 50.7–63.6).

The analysis of predicted random effects revealed that the estimates for model 2f (4Es & UHC strata) ranged from −0.76 to 0.34 with 34 of the 180 intersectional strata’s 95% CI (18.9%) not crossing zero, indicating the existence of the intersectional impacts (multiplicative effects) (Figure S3 in the **Online Supplementary Document**).

### Sensitivity analyses

The results remain consistent in the sensitivity analyses (Table S11 in the **Online Supplementary Document**). In the context of 4Es intersectional strata, the VPC increased slightly from 5.0% to 5.6% in model 2e and remains at 5% in model 3. In model 2e, the VPC also slightly increased from 90.3% to 92.1% and from 90.7% to 92.4% in model 3. The area under the curve value also increased from 61.2% to 73.0% in model 2e. The direction of the 4Es additive effects remains unchanged. In the context of 4Es combined with the national context (UHC) strata, the VPC decreased slightly from 1.0% to 0.9% in model 2e and from 0.9% to 0.8% in model 3. In model 2e, the VPC also slightly increased from 83.6% to 87.3%, remaining at 84.2% in model 3. The area under the curve value also increased from 63.1% to 73.1% in model 2e. The direction of the 4Es additive effects and UHC remained unchanged.

## DISCUSSION

Despite substantial progress in reducing MNCH mortality worldwide, significant inequities persist in the coverage of interventions, particularly in SSA [52]. This study examined the combined effect of the 4Es on MNCH intervention co-coverage across 25 SSA countries and explored how the national context influences these dynamics using the UHC index. This study aimed to determine whether combining the 4Es affects MNCH intervention co-coverage inequalities and whether these inequalities are multiplicative, additive, or both. The findings suggest that women-child pairs are more likely to receive ≥6 interventions if the women are educated (at least to primary level), not currently employed, and in better socioeconomic positions. Women’s empowerment showed no association with the co-coverage of ≥6 interventions among married women with children aged 12 and 59 months in SSA.

Our findings contrast with many studies that have reported a positive association between women’s empowerment, particularly decision-making, and the use of maternal and child health care services [53,54]. However, in their study in Sierra Leone, Sserwanja and colleagues [55] found no association between decision-making and health facility use during childbirth, which is consistent with our findings. This may be explained by the complexity of decision-making processes and their dependence on contextual factors [55]. In many SSA countries, patriarchal norms still dominate [56], and women’s autonomy in health care decisions is often limited by their partners, regardless of their perceived empowerment. Religion may also significantly influence women's decision-making autonomy, contributing to non-association [57].

While previous studies have primarily investigated the individual associations between the 4Es and MNCH service use, our study focussed on assessing the joint effects of these socioeconomic factors via an intersectional approach. The analysis revealed that the 4Es factors significantly explained disparities in MNCH co-coverage, accounting for over 90% of the between-strata variance. While the inclusion of the national UHC index slightly increased the variance, the additive effects of the 4Es remained the dominant contributors to inequalities. To ensure the robustness of these findings, we conducted sensitivity analyses considering covariates like women’s characteristics and countries. These analyses showed that adjustments for covariates and country variables did not substantially alter the findings.

The analysis of the 4Es revealed that women's education and economic status were the most significant factors explaining disparities in MNCH service coverage. Educational level has been highlighted as the most significant determinant. Women with a primary or secondary education consistently achieved better co-coverage than those without. Further analysis showed that women with a secondary or higher educational level, no current employment, belonging to the rich quintile, and a low empowerment level had the highest proportion of women with co-coverage of ≥6 interventions. This suggests that while the 4Es individually influence MNCH access [14,32,58,59], not all have a significant effect when combined. These findings reinforce the pivotal role of maternal education in improving health care access, which is consistent with the results of previous studies [31,60,61]. Education particularly improves MNCH access, even with unemployment and low empowerment. This could be explained by education enhancing access to information and health literacy, enabling women to understand the importance of seeking maternal and child health services [62]. Education equips women with the knowledge to recognise health risks and make informed decisions about their and their children's well-being [63]. Its more pronounced effect could be explained by the fact that it serves as a foundational factor that amplifies the impact of the other dimensions, such as employment, economic status, and empowerment. For example, while employment and empowerment are essential, they often rely on a basic level of education to be fully effective. Thus, education occupies a central role because it provides the necessary tools for women to make health-related decisions and access services. It acts as a baseline for the impact of other socioeconomic factors, making it the most influential dimension in improving MNCH access.

In the national context, women’s educational levels and the strength of a country’s health system, as reflected by the UHC index, were the primary contributors to inequalities in health care access. Women in countries with higher UHC indices consistently accessed more MNCH services, even without formal education. Moreno-Serra et al. stated that broader health coverage generally leads to better access to necessary care and improved population health, particularly for poor people [64]. These findings underscore the importance of individual education and national health systems in improving maternal and child health outcomes. Variations in health care system quality and accessibility across countries are critical in shaping disparities in MNCH intervention uptake, as captured by the UHC index. The UHC index provides a valuable proxy for measuring health system performance, including service availability, quality, and affordability. Healthcare system quality and accessibility may act as either enablers or barriers to MNCH service use, depending on the country context. In countries with stronger health systems, even women with lower education or economic status may benefit from widely available, accessible, and affordable MNCH services. Conversely, in countries with weaker health systems – characterised by shortages of skilled health workers, geographic inaccessibility, lack of medical supplies, and poor service quality – women face systemic barriers that can exacerbate the inequities highlighted in this study. For example, even women with higher education or economic resources may struggle to access timely and high-quality care in contexts where health infrastructure is insufficient or poorly distributed. Stronger health care systems can help offset the disadvantages of lower educational levels, ensuring broader access to essential health care services.

One of the key objectives of using the MAIHDA approach is to ascertain whether inequalities in social context are multiplicative, additive, or a combination of both. The PCV values indicated that the effects of the 4Es on inequality are primarily additive. However, certain strata displayed interaction effects, resulting in positive and negative deviations from the expected proportions of women achieving a co-coverage of ≥6. These deviations suggest that the combined impact of social determinants does not always follow a simple additive pattern. Instead, certain intersectional disadvantages may reinforce or mitigate the effects of the 4Es in ways that an additive model alone cannot fully capture. Sensitivity analyses were conducted by adjusting models for the country variable to validate these findings further. These analyses confirmed that while interaction effects exist, the additive effects remain the dominant drivers of inequalities, reinforcing the study's primary findings. Despite their socioeconomic advantages and disadvantages, women may encounter barriers that hinder their access to or use of these services. To better understand this discrepancy, further research is required to explore regional disparities and sociocultural dynamics. Addressing these additional factors could offer a more holistic approach to improving MNCH service coverage across various social strata. Analysis of the co-coverage components revealed that the inequalities between strata were most pronounced for SBA and ANC, with skilled providers and access to improved water sources. Even though there are differences in the intersectional variables, this finding aligns with that of Shibre and colleagues [65], who reported intersectional inequalities in the uptake of SBAs in Ethiopia.

Despite significant urbanisation in SSA, which has led to notable improvements in MNCH, inequalities remain prevalent within countries. For instance, while Kenya, Ghana, Tanzania, and Burkina Faso have achieved acceptable uptake of essential MNCH services, substantial disparities persist among different social strata in these countries. This indicates that progress in health coverage has not been uniform, underscoring the need for targeted interventions to address inequalities and ensure equitable access to health care services across all strata. Disparities were observed across specific interventions. For instance, in Kenya, high variability between strata was observed for six out of eight interventions, including ANC1, SBA, BCG vaccination, DPT3 vaccination, MSL vaccination, and access to improved water sources. A low predicted prevalence of each indicator was observed in the stratum with multiple socioeconomic disadvantages (no education, no employment, poorest wealth quintile, and low empowerment). This finding is consistent with the research of Mutua et al., who highlighted similar inequalities in immunisation coverage among the most disadvantaged populations in Nairobi [66]. This underscores the persistent challenges marginalised groups face in accessing essential health services, targeting the need to ensure equity across all socioeconomic clusters. An explanation to the high heterogeneity may be the disparities in the health system. Keats et al. highlighted the fact that fully functioning health systems (proper infrastructure, sufficient health workforce, consistent supply of medicines and commodities) are unequally distributed across Kenya, with poor and marginalised communities experiencing greater challenges to access MNCH services because of logistical (geographical) and financial barriers [67]. Addressing challenges to access MNCH services is imperative to achieve a widespread impact across all counties.

Eleven countries – Burundi, Zimbabwe, Zambia, Sierra Leone, Malawi, South Africa, Uganda, Gambia, Liberia, Mauritania, and Gabon – exhibited very low disparities in access to MNCH services, indicating a relatively equitable distribution of health care interventions across socioeconomic groups. Despite the ongoing political challenges, our study found that seven out of ten women in union had access to each of the eight MNCH interventions. Several factors may explain these positive results: the implementation of free medical care for pregnant women and children under five in 2006 [68,69], the introduction of health insurance for the informal sector [69]; and the scaling-up of the results-based financing approach in 2010 [70], that likely played a crucial role in improving health care access. Additionally, a study has shown that political instability in Burundi has not significantly hindered MNCH intervention coverage, with findings indicating that women in highly high-conflict regions were more likely to use skilled birth attendance services and deliver in hospitals [71]. This suggests that the relatively low heterogeneity in MNCH coverage observed in Burundi may be due to equal access to these specific services across different geographic areas, regardless of a woman's place of residence and the political context of the region. Another possible reason for the relatively high MNCH coverage in Burundi is that our study focussed exclusively on women in union, who are generally more advantaged regarding access to maternal and child health care services. A 2021 study found that MNCH service use in Burundi is primarily driven by legal marital status [72]. As a result, our findings may not fully capture the disparities single women face, who might experience more significant barriers to health care. Future research focussing on unmarried women could help better understand health care inequities and identify gaps in MNCH service coverage in this population. In Zimbabwe, another country with low heterogeneity, the results-based financing programme was introduced in 2011 and gradually expanded to all national districts by 2014, which helped substantially reduce maternal health inequalities. Makate et al. revealed a more pronounced effect of the programme on prenatal care, especially in lower socioeconomic groups, highlighting the RBF’s potential to promote equitable health care access [73].

While these results suggest positive progress toward health care equity, significant gaps remain in specific interventions in some countries with low heterogeneity in MNCH coverage. Notably, Gabon, Liberia, and South Africa demonstrated low coverage of key services such as DPT3 and MSL vaccinations. In South Africa, additional gaps were found in tetanus and BCG vaccination coverage, and Gabon showed a low uptake of Vitamin A supplementation. These findings highlight that while equitable access may have been broadly achieved, targeted efforts are still required to address these gaps. Addressing these deficiencies is the key to ensuring that improvements in equity translate into comprehensive and adequate health care coverage.

This study is the first to examine the interplay of these four key social dimensions, providing valuable insights into existing disparities in MNCH service co-coverage in SSA. By exploring the intersectionality of these dimensions, our research offers additional layers of understanding of the existing knowledge base on health care inequities in a region. The findings of this study have significant implications for targeted interventions aimed at addressing health care disparities and improving maternal health care coverage among marginalised women in SSA. Specifically, our findings underscore the importance of directing attention towards women facing multiple conditions, such as no educational level, no current employment, low empowerment level, poverty, and residing in countries with a low UHC index. By identifying these vulnerable social strata, our study highlights the need for tailored interventions that address the complex interplay of the 4Es factors contributing to inequities in maternal and child health care access and utilisation. It may also be used to support more targeted resources, programmes, and policies to reduce the risk of low utilisation of MNCH services among women with these social identities.

Our study had several limitations. First, the social strata used were based on the 4Es, selected for their strong associations with the outcome. While this allowed for an initial intersectionality analysis of MNCH co-coverage in SSA, future studies should include additional factors, such as subnational and cultural disparities, to assess inequities better. The PROGRESS framework includes other dimensions that may be explored in the future [26]. In this study, we could not investigate subregional differences due to the divergence in administrative divisions across the 25 countries. We, therefore, suggest that future studies conduct regional-level investigations in countries with high heterogeneity to capture local-level variations that could inform targeted regional interventions. This approach would allow a deeper understanding of how regional disparities contribute to health care inequities. Second, the co-coverage calculations relied on self-reported mother data, which may have introduced recall bias. To minimise the impact of this bias, we exclusively considered the last live birth for the analysis. However, residual bias could have persisted but does not affect the consistency of the results. Third, the study included only married women, which could limit the generalisability of the findings to single mothers. We limited our study to this population because the empowerment indicators (decision-making within the household and attitudes toward wife-beating) were collected only among married women. However, although the proportion of single mothers is increasing in Africa, it remains low, varying from 10% in Nigeria to 30% in Zimbabwe [74]. This relatively low prevalence suggests that the population considered in our study remains slightly representative of most women using MNCH services in the region, reinforcing the validity of our findings by limiting the bias. Future research could focus on this population to capture their unique experiences and ensure the broader generalisability of findings. Another limitation of our study is the narrow operationalisation of women's empowerment, which was defined based on decision-making autonomy and attitude toward wife-beating. While these indicators are widely used in global health research [75,76] and align with established frameworks, empowerment is a complex and multidimensional construct encompassing broader aspects [77]. Our findings, therefore, are conservative in capturing the extent of inequalities. Due to the multiplicity of definitions, we adhered to the DHS definition to ensure consistency and comparability across countries. However, future research should consider incorporating additional dimensions of empowerment to provide a more comprehensive understanding of its impact on MNCH service use and health outcomes. Our study offers an initial evaluation of intersectional differences in MNCH coverages. It can be used as a starting point for deeper analysis, considering broader social dimensions such as structural and health care accessibility factors (*e.g.* distance to health care facilities, transportation and road infrastructure, travel costs), socio-cultural factors (*e.g.* family support, perception of pregnancy-related issues as a private matter for women, relationships with traditional birth attendants in the community), individual factors (*e.g.* high parity, lack of birth preparedness plans) and religious factors [78–81]. While MAIHDA reduces the risk of misclassification bias by considering multiple intersecting social determinants simultaneously, it does not eliminate the influence of unmeasured confounders, such as health system factors, cultural norms, or religious factors.

## CONCLUSIONS

We explored the intersectional effects of the 4Es on MNCH intervention co-coverage in SSA using a large-scale data set from 25 countries. The findings provide robust evidence of significant disparities in MNCH service coverage linked to these social factors, with educational levels emerging as the most critical determinant. Additionally, this study highlights the role of the national context, particularly the UHC index, in shaping these inequalities. These results underscore the need for a multidimensional approach to address inequities in MNCH services in SSA, considering the intersecting influences of multiple social determinants and the broader national context. Policies to increase women’s educational levels and strengthen national health systems can significantly reduce disparities in these services and improve health outcomes for mothers and children in the region. Specifically, tailored health awareness programmes may enhance the understanding and use of MNCH services through community health workers who deliver these programmes in local languages, especially in rural areas. Moreover, policies may focus on removing out-of-pocket expenses for essential MNCH services by integrating them into UHC schemes. Further research is needed to explore the patterns of these intersectional effects, considering other social dimensions, and assess these inequalities' temporal trends. This is crucial for monitoring the progress of structural strategies aimed at reducing disparities across the countries of SSA.

## Additional material


Online Supplementary Document
