# Supplementary material for: Assessing the joint effects of education, economic status, empowerment, and employment (4Es) disparities on the co-coverage of maternal, newborn and child health care services in sub-Saharan Africa: an application of the intersectionality approach
Source: J Glob Health. 2025 May 9;15:04124. doi: 10.7189/jogh.15.04124 (PMC12063645; doi:10.7189/jogh.15.04124)
Supplement: Online Supplementary Document [file jogh-15-04124-s001.pdf]

Supplement to: Kirakoya-Samadoulougou F, Fassinou LC, Garba MLI, Maïga A, Zeger SL, Amouzou A. Assessing the joint effects of education, economic status, empowerment, and employment (4Es) disparities on the co-coverage of maternal, newborn and child healthcare services in sub-Saharan Africa: an application of the intersectionality approach. J Glob Health. 2025; 15:04124.

Supplementary material 1

1. **Table S1. Description of the study population.**

N°	Country	Year of survey	Sample size	Women included (N)	Weighted N
1	Angola	2015-2016	14,379	3,942	3,903
2	Benin	2017-2018	15,928	5,182	5,173
3	Burkina Faso	2021	17,659	3,625	3,624
4	Burundi	2016-2017	17,269	4,993	5,158
5	Cameroon	2018-2019	14,677	3,129	3,323
6	Ivory Coast	2021	14,977	2,656	2,524
7	Ethiopia	2016	15,683	4,334	4,597
8	Gabon	2019	11,043	1,889	1,878
9	Gambia	2019-2020	11,865	3,390	3,103
10	Ghana	2022	15,014	2,665	2,347
11	Guinea	2018	10,874	3,333	3,303
12	Kenya	2022	32,156	4,731	4,289
13	Liberia	2019-2020	8,065	2,008	1,770
14	Madagascar	2021	18,869	5,006	5,016
15	Malawi	2015-2016	24,562	7,939	8006
16	Mali	2018	10,519	3,929	4,093
17	Mauritania	2019-2021	15,714	4,406	4,461
18	Nigeria	2018	41,821	12,993	13,175
19	Rwanda	2019-2020	14,634	3,552	3,625
20	Sierra-Leone	2019	15,574	4,058	3,977
21	Tanzania	2022	15,524	2,754	2,808
22	South Africa	2016	8,514	905	1,007
23	Uganda	2016	18,506	5,377	5,250

24	Zambia	2018-2019	13,683	3,781	3,822
25	Zimbabwe	2015	9,955	2,811	2,947

2. Table S2. Definition of variables used to construct the social strata

N°	Variable	Categories	Definition
1	Education	-no education<br>-primary<br>-secondary/higher	The highest level of school they attended, and the highest grade they completed at that level.
2	Employment	-not currently employed<br>-currently employed	Whether the respondent is currently employed (have done any work in the last 12 months) or not
3	Economic status	-poorest<br>-poorer<br>-middle<br>-richer<br>-richest	Quintiles of the wealth index measuring detailed information on dwelling, household characteristics, access to a variety of consumer goods and services and assets.
4	Empowerment	-low<br>-middle<br>-high	Woman making all three specific decisions (own healthcare, large household purchases, visits to family or relatives) either alone or jointly with her husband.
5	Universal health coverage	-low<br>-high	The capacity of the country to offer access to the full range of quality health services they need, when and where they need them, without financial hardship.

3. Table S3. List of skilled healthcare professional per country

N°	Country	Skilled professional	Non-skilled professional
1	Angola	- Doctor<br>- Nurse<br>- Midwife<br>- Health professionals	- Traditional birth attendant<br>- Itinerant health worker

2	Benin	- Doctor<br>- Nurse<br>- Midwife<br>- Auxiliary nurse<br>- CS health professional<br>- Auxiliary midwife (matron)	- Traditional birth attendant<br>- Friends/relative<br>- CS other<br>- Other
3	Burkina Faso	- Doctor<br>- Health advisors<br>- Midwife<br>- Nurse<br>- Certified birth attendant<br>- Auxiliary birth attendant	- Traditional birth attendant<br>- Itinerant health worker<br>- Community health workers<br>- CS other persons<br>- other
4	Burundi	- Doctor<br>- Midwife<br>- Nurse<br>- CS health professional	- Traditional birth attendant<br>- Community health worker<br>- Other
5	Ivory coast	- Doctor<br>- Nurse/midwife<br>- Auxiliary midwife<br>- CS heath professional	- Traditional birth attendant<br>- Community health worker<br>- Parents/friend<br>- CS other persons<br>- other
6	Cameroon	- Doctor<br>- Nurse/midwife<br>- Auxiliary midwife<br>- CS health professional<br>-	- Relative/friends<br>- CS other<br>- other
7	Ethiopia	- Doctor<br>- Nurse<br>- Midwife<br>- Health officer	- Health extension<br>- Family/friends<br>- Traditional birth attendant
8	Gabon	- Doctor (gynecologist)<br>- Others doctor<br>- Midwife<br>- Nurse acoucher<br>- Nurse<br>- CS health professional	- Traditional birth attendant<br>- Relative/friends

		- Matron	
9	Ghana	- Doctor<br>- Nurse/midwife<br>- CS health professional	- Traditional birth attendant<br>- Community health worker
10	Gambia	- Doctor<br>- Nurse/midwife<br>- Auxiliary nurse/	
11	Guinea	- Doctor<br>- Nurse/midwife<br>- Technical health officer	- Traditional birth attendant<br>- Community/village health worker
12	Kenya	- Doctor<br>- Nurse/midwife<br>- Clinical officer	- Traditional birth attendant<br>- other
13	Liberia	- Doctor<br>- Nurse/midwife<br>- Physician assistant<br>-	- Traditional birth attendant<br>- Other
14	Madagascar	- Doctor<br>- Nurse<br>- Midwife	- Traditional birth attendant<br>- Other
15	Mali	- Doctor<br>- Nurse/midwife<br>- Matron<br>- CS health professional	- Trained traditional birth attendant<br>- Community health worker<br>- Relative/friend<br>- Other
16	Mauritania	- Doctor<br>- Nurse/midwife<br>- Auxiliary midwife<br>- CS health professional	- Traditional birth attendant<br>- Community health worker<br>- Friends/relative<br>- Other
17	Malawi	- Doctor/clinical officer/medical assistant<br>- Nurse/midwife<br>- CS health professional	- Traditional birth attendant<br>- Patient attendant<br>- Relative/friends<br>- Other
18	Nigeria	- Doctor<br>- Nurse/midwife<br>- Auxiliary midwife	- Community health extension worker<br>- Traditional birth attendant<br>- Relative/friends

19	Rwanda	- Doctor<br>- Nurse/midwife<br>- Auxiliary midwife<br>-	- Traditional birth attendant<br>- Community health worker<br>- Relative/friends<br>- Other
20	South Africa	- Doctor/gynecologist<br>- Nurse/midwife	- Traditional birth attendant<br>- Relative/friends<br>- Other
21	Sierra Leonne	- Doctor<br>- Nurse/midwife<br>- Auxiliary midwife	- Traditional birth attendant<br>- Relative/friends<br>- Other
22	Tanzania	- Doctor/assistant medical officer<br>- Clinical officer<br>- Assistant clinical officer<br>- Nurse/midwife<br>- Assistant nurse<br>- Maternal and child aide	- Traditional birth attendant<br>- Community health workers<br>- Relative/friends<br>- Other
23	Uganda	- Doctor<br>- Nurse/mid<br>- Medical assistant/clinical officer<br>- Nursing aid/assistant<br>- CS health professional	- Traditional birth attendant<br>- Relative/friends<br>- Other
24	Zambia	- Doctor`<br>- Nurse/mid<br>- Clinical officer<br>- CS health professional	- Traditional birth attendant<br>- Community health worker<br>- Relative/friends
25	Zimbabwe	- Doctor`<br>- Nurse/mid<br>- Clinical officer<br>- CS health professional	- Traditional birth attendant<br>- Community health worker<br>- Relative/friends<br>-

4. **Table S4. Prevalence of each MNCH indicator per country among women and children in sub-Saharan Africa 2015-2022 with observations clustered by 4Es-social strata (weighted N= 103,178).**

Country	ANC1 with skilled provider	TT	SBA (%; 95%CI)	Vit A	BCG	DPT3	MSL	Improved water source (%; 95%CI)
Angola	81.2 (78.6-83.9)	68.4 (65.4-71.4)	54.3 (50.6-57.9)	4.9 (3.9-5.9)	54.8 (52.1-57.6)	29.1 (26.7-31.5)	44.1 (41.3-46.8)	68.3 (64.1-72.5)
Benin	86.2 (84.3-88.0)	75.1 (72.9-77.2)	82.1 (79.6-84.7)	55.4 (52.9-58.0)	63.2 (61.4-64.9)	53.0 (51.1-55.0)	49.4 (47.4-51.4)	68.0 (64.8-71.3)
Burkina Faso	97.6 (96.8-98.4)	67.7 (65.3-70.1)	95.5 (94.2-96.9)	43.8 (41.2-46.4)	96.7 (95.7-97.6)	88.6 (86.8-90.4)	86.9 (85.3-88.4)	81.2 (78.1-84.4)
Burundi	99.4 (99.1-99.6)	82.5 (81.1-84.0)	90.0 (88.9-91.2)	75.9 (74.0-77.7)	73.0 (71.6-74.4)	72.4 (71.0-73.9)	70.6 (69.2-72.0)	82.1 (79.7-84.4)
Cameroon	85.5 (83.1-87.8)	71.5 (68.6-74.5)	68.4 (63.7-73.0)	57.0 (54.2-59.8)	60.7 (58.2-63.2)	50.5 (47.7-53.2)	48.1 (45.5-50.8)	71.6 (67.3-75.9)
Ivory Coast	94.2 (93.0-95.5)	69.4 (66.8-72.0)	83.8 (81.3-86.3)	51.3 (47.5-57.2)	87.6 (85.6-89.6)	54.4 (50.9-57.9)	62.0 (59.0-65.0)	82.1 (79.3-85.0)
Ethiopia	60.8 (57.3-64.2)	52.0 (48.9-55.2)	27.9 (24.6-31.3)	51.6 (48.8-54.4)	46.3 (43.9-48.9)	34.9 (32.2-37.6)	36.9 (34.2-39.6)	60.4 (55.8-65.0)
Gabon	91.8 (89.0-94.6)	78.3 (74.6-81.9)	96.3 (95.2-97.3)	22.1 (18.9-25.3)	57.1 (53.1-61.1)	43.1 (39.2-46.9)	44.2 (40.0-48.5)	94.1 (92.5-95.6)
Gambia	98.9 (98.5-99.4)	65.8 (63.2-68.3)	85.2 (83.0-87.3)	65.2 (62.3-68.0)	68.2 (65.9-70.4)	64.1 (61.8-66.4)	62.8 (60.3-65.4)	93.8 (91.4-96.2)
Ghana	97.8 (97.0-98.6)	71.9 (69.2-74.6)	86.8 (84.0-89.5)	85.1 (83.1-87.0)	94.9 (93.7-96.0)	89.4 (87.7-91.0)	88.9 (87.2-90.7)	85.1 (82.1-88.1)
Guinea	77.8 (75.1-80.5)	58.8 (55.8-61.8)	56.3 (52.5-60.2)	43.4 (40.5-46.3)	47.5 (44.8-50.1)	26.5 (24.1-29.0)	27.2 (24.5-29.8)	76.9 (73.4-80.4)
Kenya	98.1 (97.7-98.6)	69.1 (67.1-71.0)	88.8 (87.5-90.1)	76.7 (74.7-78.6)	97.3 (96.7-97.9)	89.6 (88.4-90.8)	89.7 (88.5-90.9)	77.0 (74.9-79.0)
Liberia	96.5 (95.2-97.7)	85.7 (83.1-88.2)	85.5 (82.9-88.0)	50.8 (46.6-55.0)	54.6 (51.9-57.3)	41.1 (38.2-43.9)	44.6 (41.8-47.5)	81.9 (78.0-85.9)
Madagascar	89.9 (88.4-91.4)	75.0 (73.1-76.9)	50.7 (47.5-53.9)	43.8 (41.3-46.3)	49.4 (47.4-51.3)	43.7 (41.7-45.6)	41.9 (39.9-43.8)	45.4 (41.4-49.3)
Malawi	94.4 (93.6-95.2)	90.6 (89.7-91.4)	90.3 (89.1-91.4)	68.5 (66.8-70.2)	60.0 (58.6-61.4)	57.2 (55.7-58.6)	56.9 (55.5-58.3)	87.2 (85.5-89.0)
Mali	76.7 (73.9-79.5)	48.2 (45.4-51.1)	70.5 (66.6-74.3)	69.2 (66.6-71.8)	61.8 (59.3-64.4)	51.9 (49.1-54.6)	51.8 (49.1-54.5)	68.6 (64.4-72.8)
Mauritania	78.8 (76.0-81.5)	57.9 (55.5-60.3)	74.1 (70.4-77.9)	64.5 (61.7-67.4)	63.0 (60.8-65.1)	50.9 (48.5-53.3)	53.4 (51.0-55.7)	77.8 (73.9-81.6)
Nigeria	71.4 (69.4-73.4)	60.1 (58.2-62.1)	45.2 (42.9-47.5)	48.4 (46.3-50.5)	45.9 (44.2-47.6)	34.0 (32.4-35.6)	38.8 (37.3-40.3)	69.9 (67.3-72.4)
Rwanda	98.2 (97.7-98.7)	74.3 (72.6-76.0)	93.5 (92.4-94.6)	90.3 (89.0-91.5)	63.1 (61.4-64.8)	62.9 (61.1-64.6)	62.1 (60.4-63.8)	78.8 (75.9-81.7)

Sierra-Leone	86.2 (83.9-88.6)	83.9 (82.1-85.7)	87.6 (85.7-89.5)	70.9 (68.7-73.0)	62.9 (61.2-64.5)	50.5 (8.7-52.4)	51.2 (49.4-53.0)	61.9 (57.9-65.8)
Tanzania	88.3 (86.5-90.1)	86.2 (84.4-88.1)	85.7 (83.1-88.3)	61.9 (58.9-64.9)	91.5 (89.9-93.1)	90.7 (89.0-92.5)	88.5 (86.6-90.4)	68.4 (64.3-72.5)
South Africa	90.1 (87.2-92.9)	49.3 (44.5-54.1)	96.3 (94.8-97.8)	74.2 (70.6-77.8)	48.2 (44.1-52.4)	31.9 (28.4-35.5)	45.4 (41.3-49.6)	96.0 (94.3-97.7)
Uganda	97.4 (96.7-98.0)	79.8 (78.4-81.3)	76.7 (74.7-78.7)	66.4 (64.5-68.3)	66.6 (65.2-68.0)	54.8 (53.2-56.4)	57.8 (56.1-59.4)	78.2 (75.7-80.8)
Zambia	96.2 (95.4-97.1)	76.6 (74.7-78.4)	81.7 (79.6-83.9)	78.4 (76.4-80.3)	65.5 (63.6-67.3)	61.8 (59.8-63.8)	61.9 (59.9-63.9)	70.3 (66.7-73.9)
Zimbabwe	93.7 (92.2-95.2)	53.5 (50.8-56.1)	79.5 (76.8-82.3)	72.1 (69.2-75.0)	56.0 (53.8-58.2)	52.5 (50.4-54.7)	52.4 (50.2-54.7)	75.4 (71.4-79.3)

%– proportion, CI – confidence interval.

5. **Table S5. sample size of 4Es and country-level (UHC) social strata defined as the combination of maternal education, economic status, empowerment, and employment.**

**Sample size**	**4Es-social strata (N=90)**	**4Es and UHC social strata (N=180)**
	Number of strata (%)	Number of strata (%)
1-100	0	0
101-500	22	11
501-1,000	30	94
1,001-5,000	38	48
5,001-10,000	0	27
10,001-103,388	0	0

6. **Table S6. Estimates from multilevel models of each component of co-coverage among women and children in sub-Saharan Africa 2015-2022 with observations clustered by 4Es-social strata (N= 103,388).**

	ANC	Tetanus vaccination	SBA	Vitamin A	BCG	DPT3	MSL	Improved water source
VPC (empty model) (%)	14.5	4.8	15.3	2.6	2.0	2.7	2.3	10.9
VPC (adjusted model for 4E) (%)	0.7	0.2	1.0	0.2	0.4	0.4	0.3	0.6
PCV (%)	95.5	96.0	94.2	93.0	80.8	86.9	88.6	95.4
AUC (%)	72.0	70.9	70.9	70.9	57.2	58.3	56.8	54.6
Lowest predicted proportion (%, 95%CI)	63.0 (59.4-66.5)	44.4 (42.0-46.9)	35.8 (31.6-40.4)	40.1 (37.5-42.8)	50.3 (47.1-53.3)	37.1 (34.4-40.0)	40.7 (38.1-44.0)	51.5 (48.5-54.6)
Highest predicted proportion (%, 95%CI)	96.6 (95.6-97.4)	81.9 (79.6-84.1)	94.3 (93.2-95.1)	70.0 (67.1-72.7)	73.7 (70.5-76.9)	64.0 (60.7-67.5)	65.5 (62.1-68.5)	94.4 (93.0-95.5)
Stratum* with lowest predicted proportion (%, 95%CI) *	1111	1112	1113	1111	1111	1111	1112	1112
Stratum* with highest predicted proportion (%, 95%CI) *	3253	3253	3252	2253	3141	3151	3141	3151

%– proportion, CI – confidence interval.

*The four dimensions (4Es) of the strata are presented in the following order: educational level: 1=no education, 2=primary, 3=secondary/higher; employment: 1=not currently employed, 2= currently employed; economic status: 1= poorest, 2=poorer, 3=middle, 4=richer, 5=richest; and empowerment: 1=low, 2=middle, 3=high.

7. **Table S7. Estimates from multilevel models of co-coverage with at least six interventions among women and children per country**

	Angola	Benin	Burkina Faso	Burundi	Cameroon	Ivory Coast	Ethiopia	Gabon	Gambia
VPC (empty model) (%)	18.6	6.3	11.9	0.4	9.1	9.5	13.8	4.9	1.8
VPC (adjusted model for 4E) (%)	0.5	0.0	0.02	0.0	0.0	0.0	0.4	0.7	0.0
PCV (%)	97.7	100	99.9	100	100	100	97.7	86.4	100
AUC (%)	74	64	70	56	67		73	64	60
Lowest predicted proportion (%, 95%CI)	7.9 (5.4-10.9)	27.5 (22.5-32.6)	62.2 (50.9-72.5)	60.2 (52.1-67.8)	22.3 (17.7-27.6)	40.0 (34.8-45.0)	9.1 (7.0-11.9)	23.4 (16.6-32.8)	55.0 (49.3-60.6)

Highest predicted proportion (%, 95%CI)	53.5 (43.2-63.3)	69.6 (65.4-73.5)	95.6 (93.7-97.0)	75.4 (70.8-79.1)	60.4 (55.7-65.5)	85.4 (78.6-90.5)	61.5 (53.5-69.3)	52.1 (41.3-62.4)	76.7 (72.3-81.2)
Stratum* with lowest predicted proportion (%, 95%CI)	1211	1113	1113	1153	1213	1112	1113	2113	1223
Stratum* with highest predicted proportion (%, 95%CI)	3153	3242	3242	3242	3152	3243	3232	3251	3142

	Ghana	Guinea	Kenya	Liberia	Madagascar	Malawi	Mali	Mauritania	Nigeria
VPC (empty model) (%)	15.7	13.5	26.0	3.1	9.4	0.9	8.9	4.5	19.7
VPC (adjusted model for 4E) (%)	0.0	0.0	1.5	0.5	0.0	0.1	0.0	0.0	1.1
PCV (%)	100	100	95.7	83.0	100	92.7	100	100	95.5
AUC (%)	75	68	83	63	67	57	65	63	76
Lowest predicted proportion (%, 95%CI)	67.8 (57.4-76.7)	10.8 (8.2-14.1)	38.4 (29.0-48.2)	33.9 (27.1-41.5)	12.1 (9.7-14.9)	44.5 (38.1-51.1)	21.9 (18.1-25.7)	34.4 (29.0 -40.1)	5.7 (4.0-8.0)
Highest predicted proportion (%, 95%CI)	97.8 (95.8-98.8)	61.5 (54.2-67.9)	97.3 (95.0-98.5)	63.0 (52.5-73.2)	52.9 (46.7-58.6)	65.8 (68.0-69.6)	70.1 (65.8 - 74.2)	66.7 (62.1 - 71.0)	60.9 (52.8-68.4)
Stratum* with lowest predicted proportion (%, 95%CI) *	1113	1111	1111	1213	1211	1253	1111	1223	1112
Stratum* with highest predicted proportion (%, 95%CI) *	3252	3253	3241	3121	3143	2111	3252	3151	3152

	Rwanda	South Africa	Sierra Leone	Tanzania	Uganda	Zambia	Zimbabwe
VPC (empty model) (%)	-	1.2	0.7	19.7	1.4	0.5	0.4
VPC (adjusted model for 4E) (%)	-	0.0	0.0	0.0	0.1	0.0	0.0
PCV (%)	-	100	100	100	92.0	100	100
AUC (%)		60	57	77	58	58	57

Lowest predicted proportion (%, 95%CI)	49.3 (42.9-56.8)	19.6 (8.6-38.4)	44.9 (39.3-49.5)	43.3 (35.6-51.6)	40.8 (34.5-47.8)	43.6 (36.5-51.0)	35.8 (29.6-53.8)
Highest predicted proportion (%, 95%CI)	72.8 (66.6-78.0)	53.1 (35.8-68.5)	64.0 (57.4-70.7)	96.9 (95.1-98.0)	69.7 (64.6-74.2)	67.4 (62.8-71.2)	58.8 (53.9-63.0)
Stratum*with lowest predicted proportion (%, 95%CI) *	1253	1232	1213	1113	1241	1253	1213
Stratum* with highest predicted proportion (%, 95%CI) *	3112	3143	2151	3251	3212	3142	3122

%– proportion, CI – confidence interval.

*The four dimensions (4Es) of the strata are presented in the following order: educational level: 1=no education, 2=primary, 3=secondary/higher; employment: 1=not currently employed, 2= currently employed; economic status: 1= poorest, 2=poorer, 3=middle, 4=richer, 5=richest; and empowerment: 1=low, 2=middle, 3=high.

8. **Table S8. Estimates from multilevel models of MNCH interventions among women and children per country**

	Angola	Benin	Burkina Faso	Burundi	Cameroon	Ivory Coast	Ethiopia	Gabon	Gambia
**ANC1**									
VPC (empty model) (%)	26.1	14.9	0.94	-	23.7	10.6	18.1	12.7	0.0
VPC (adjusted model for 4E) (%)	0.0	0.0	0.0	-	0.0	0.0	0.0	0.0	0.0
PCV (%)	100	100	100	-	100	100	100	100	87.7
AUC (%)	83	72	70	50	78	71	73	75	79
Lowest predicted proportion (%, 95%CI)	92.7 (88.4-95.5)	53.5 (48.3-59.5)	93.8 (83.3-97.8)	93.7 (81.7-97.8)	56.5 (49.9-63.0)	84.0 (79.8-87.6)	37.3 (34.0-41.0)	72.4 (59.2-82.4)	96.0 (90.0-98.4)
Highest predicted proportion (%, 95%CI)	100.0	97.0 (95.1-98.1)	99.1 (97.9-99.6)	99.9 (99.6-100)	97.2 (95.2-98.3)	98.3 (96.4-99.2)2.4	94.8 (92.2-96.5)	97.1 (94.8-98.4)	99.6 (98.7-99.9)
Stratum* with lowest predicted proportion (%, 95%CI)	1211	1111	2143	1143	1111	1212	1113	1121	1133
Stratum* with highest predicted proportion (%, 95%CI)	3152	3243	3231	3252	3253	2151	3251	3243	3111
Tetanus vaccination									
VPC (empty model) (%)	7.8	7.5	1.8	0.3	12.3	2.4	13.0	2.9	1.3
VPC (adjusted model for 4E) (%)	0.0	0.0	0.2	0.0	0.0	0.0	0.3	0.0	0.0
PCV (%)	100	100	90.3	100	100	100	97.7	100	100
AUC (%)	67	67	60	57	71	61	71	64	60
Lowest predicted proportion (%, 95%CI)	37.4 (29.4-46.2)	49.5 (44.3-54.4)	57.1 (51.1-62.8)	75.7 (71.0-80.0)	38.1 (30.5-45.9)	57.1 (49.2-65.7)	25.7 (21.2-30.3)	53.4 (45.1-61.6)	53.3 (46.9-59.9)
Highest predicted proportion (%, 95%CI)	83.7 (79.3-87.4)	88.9 (86.3-91.0)	78.6 (73.3-83.3)	88.4 (83.3-92.1)	86.2 (82.5-89.0)	81.9 (76.3-86.2)	86.6 (81.1-90.8)	79.3 (74.3-83.8)	78.6 (73.8-82.8)
Stratum* with lowest predicted proportion (%, 95%CI) *	1113	1111	1112	2221	1113	1213	1113	2123	3111
Stratum* with highest predicted proportion (%, 95%CI) *	3251	3252	3252	3153	3251	3152	3251	3252	1253
Skilled birth attendant									

VPC (empty model) (%)	11.6	19.6	12.8	7.1	37.7	11.7	32.5	16.0	9.0
VPC (adjusted model for 4E) (%)	0.5	0.3	0.0	0.0	0.0	0.0	0.4	0.0	0.0
PCV (%)	95.7	98.8	100	100	100	100	99.1	100	100
AUC (%)	72	75	74	65	84	69	78	77	68
Lowest predicted proportion (%, 95%CI)	68.9 (59.0-76.9)	47.0 (39.1-55.2)	77.9 (66.6-87.0)	81.5 (77.1-85.5)	24.7 (20.2-29.4)	61.8 (57.2-66.3)	8.5 (6.6-11.3)	73.5 (65.3-80.6)	60.5 (54.7-66.3)
Highest predicted proportion (%, 95%CI)	96.8 (94.2-98.3)	95.9 (93.9-97.3)	99.1 (97.9-99.6)	99.0 (97.8-99.6)	97.4 (96.2-98.3)	97.6 (95.4-98.8)	88.8 (84.2-91.8)	97.7 (95.5-99.0)	94.5 (92.2-96.1)
Stratum* with lowest predicted proportion (%, 95%CI) *	1213	1112	1113	1211	1211	1212	1113	2122	1213
Stratum* with highest predicted proportion (%, 95%CI) *	3152	2251	3222	3153	3152	3153	3252	3251	3141
Vitamin A supplementation									
VPC (empty model) (%)	3.5	2.7	2.1	1.0	1.0	3.8	3.6	1.6	0.5
VPC (adjusted model for 4E) (%)	0.0	0.0	0.2	0.0	0.0	0.0	0.5	0.0	0.0
PCV (%)	100	100	89.0	100	100	100	86.4	100	100
AUC (%)	63	60	60	58	60	62	63	65	59
Lowest predicted proportion (%, 95%CI)	69.5 (57.0-79.3)	34.7 (29.2-40.4)	35.0 (26.8-44.9)	52.2 (45.0-60.1)	36.2 (30.7-43.3)	32.1 (27.7-37.5)	35.9 (30.6-42.3)	9.2 (6.2-13.1)	58.0 (51.9-63.2)
Highest predicted proportion (%, 95%CI)	91.7 (88.4-94.0)	66.0 (61.8-70.2)	55.5 (48.1-62.7)	84.3 (79.8-87.6)	65.6 (59.4-71.4)	70.3 (61.4-78.7)	73.7 (65.2-81.0)	24.4 (18.0-32.4)	74.0 (68.5-78.6)
Stratum* with lowest predicted proportion (%, 95%CI) *	1143	1113	1123	1152	1142	1112	1113	2112	1223
Stratum* with highest predicted proportion (%, 95%CI) *	3231	2251	3231	3213	3223	2253	3241	3221	2112
BCG vaccination									
VPC (empty model) (%)	6.3	4.1	22.5	0.45	1.4	12.2	4.7	0.7	1.6
VPC (adjusted model for 4E) (%)	0.0	0.0	0.0	0.0	0.0	0.0	0.5	0.0	0.0
PCV (%)	100	100	100	100	100	100	90.0	100	100

AUC (%)	69	62	80	56	60	69	64	61	60
Lowest predicted proportion (%, 95%CI)	85.4 (76.7-91.5)	45.9 (40.5-51.9)	87.9 (82.8-91.4)	60.5 (52.6-67.5)	47.8 (42.7-52.9)	73.3 (67.6-78.2)	27.8 (22.9-32.9)	45.1 (36.2-55.7)	60.6 (54.7-66.0)
Highest predicted proportion (%, 95%CI)	97.3 (95.5-98.5)	77.8 (73.4-81.9)	99.7 (98.7-99.9)	77.4 (73.2-81.2)	68.9 (63.7-73.4)	97.5 (94.5-98.7)	66.3 (58.3-74.1)	61.5 (55.1-67.6)	78.9 (73.3-83.4)
Stratum[*] with lowest predicted proportion (%, 95%CI) *	1113	1213	1111	1153	1212	1112	1113	1241	1233
Stratum[*] with highest predicted proportion (%, 95%CI) *	3242	3132	2243	2231	3231	3243	3132	3152	2141
DPT3 vaccination									
VPC (empty model) (%)	9.7	4.6	8.3	0.27	3.0	4.1	7.3	0.4	1.1
VPC (adjusted model for 4E) (%)	3.3	0.0	0.6	0.0	0.0	0.0	0.7	0.0	0.0
PCV (%)	68.4	100	93.5	100	100	100	90.1	100	100
AUC (%)	68	62	68	56	61	63	68	60	60
Lowest predicted proportion (%, 95%CI)	67.7 (52.2-80.6)	36.1 (30.3-42.0)	75.8 (68.3-81.7)	61.0 (53.6-67.9)	35.4 (30.2-41.1)	38.7 (33.9-43.9)	15.8 (12.4-20.3)	32.2 (23.1-43.3)	56.3 (51.1-61.7)
Highest predicted proportion (%, 95%CI)	93.6 (87.1-97.0)	69.4 (64.2-74.0)	94.9 (91.9-96.8)	76.5 (73.2-79.4)	59.8 (54.4-64.9)	76.4 (67.9-82.9)	60.4 (49.0-70.4)	51.0 (44.5-57.3)	74.9 (69.3-79.4)
Stratum[*] with lowest predicted proportion (%, 95%CI) *	1111	1113	1111	1153	1112	1111	1113	1143	1223
Stratum[*] with highest predicted proportion (%, 95%CI) *	3233	3142	2242	2222	3231	2243	3221	3152	2141
MSL vaccination									
VPC (empty model) (%)	6.6	3.0	5.7	0.5	1.8	3.2	4.9	2.7	1.3
VPC (adjusted model for 4E) (%)	0.0	0.0	0.03	0.0	0.0	0.0	0.2	0.1	0.1
PCV (%)	100	100	99.5	100	100	100	96.5	94.6	95.6
AUC (%)	68	60	66	56	60	62	65	63	60
Lowest predicted proportion (%, 95%CI)	72.3 (63.0-80.5)	36.4 (32.4-40.6)	74.7 (63.3-82.5)	56.1 (49.1-63.7)	34.2 (27.8-41.4)	50.2 (44.9-55.6)	24.6 (20.8-28.8)	26.9 (20.2-34.2)	57.0 (51.0-62.7)

Highest predicted proportion (%, 95%CI)	94.3 (91.0-96.5)	63.6 (59.2-68.1)	94.5 (91.9-96.2)	75.7 (71.6-79.7)	56.2 (50.8-61.5)	81.3 (76.4-85.7)	61.3 (52.7-69.1)	48.3 (40.2-56.4)	73.3 (67.9-78.1)
Stratum* with lowest predicted proportion (%, 95%CI) *	1113	1111	1133	1153	1113	1111	1113	2113	1223
Stratum* with highest predicted proportion (%, 95%CI) *	3251	3232	3241	2221	3251	3252	3221	3231	3142
Access to improved water source									
VPC (empty model) (%)	19.6	11.0	6.6	5.7	12.8	9.7	15.0	12.1	16.6
VPC (adjusted model for 4E) (%)	0.0	0.0	0.0	0.0	0.5	0.0	0.0	0.0	1.5
PCV (%)	100	100	100	100	96.8	100	100	100	92.2
AUC (%)	76	68	64	63	71	69	70	72	75
Lowest predicted proportion (%, 95%CI)	54.0 (48.3-59.3)	39.9 (35.5-44.5)	69.9 (65.3-74.4)	74.5 (70.7-77.8)	45.4 (35.3-55.6)	57.5 (46.4-67.8)	38.7 (33.4-44.7)	53.3 (45.3-62.0)	83.8 (73.1-90.0)
Highest predicted proportion (%, 95%CI)	98.3 (96.7-99.2)	88.9 (86.3-90.9)	94.9 (91.6-96.9)	95.7 (93.3-97.4)	91.0 (87.5-93.8)	93.8 (90.5-96.0)	94.6 (92.3-96.2)	94.1 (91.3-96.1)	99.0 (97.7-99.6)
Stratum* with lowest predicted proportion (%, 95%CI) *	1211	1112	1111	1223	1223	2213	1111	2113	1211
Stratum* with highest predicted proportion (%, 95%CI) *	3151	3251	3253	3152	3151	3142	3253	3252	3142

	Ghana	Guinea	Kenya	Liberia	Madagascar	Malawi	Mali	Mauritania	Nigeria
**ANC1**									
VPC (empty model) (%)	26.1	7.9	35.1	-	27.9	1.3	17.5	11.4	16.1
VPC (adjusted model for 4E) (%)	0.0	0.0	6.2	-	0.02	0.1	0.6	0.0	1.1
PCV (%)	100	100	87.8	-	99.9	91.6	97.2	100	94.0
AUC (%)	83	64	87	50	77	62	71	69	75
Lowest predicted proportion (%, 95%CI)	92.7 (88.4-95.5)	62.1 (56.5-67.5)	81.9 (62.8-92.3)	90.5 (83.9-94.5)	63.9 (55.5-71.5)	89.5 (85.0-92.7)	40.1 (32.6-49.2)	61.5 (55.6-67.1)	30.2 (25.1-36.5)
Highest predicted proportion (%, 95%CI)	100.0	93.6 (91.0-95.6)	99.8 (98.8-100.0)	97.7 (94.4-98.9)	98.5 (97.7-99.1)	97.3 (96.1-98.2)	96.3 (94.2-97.7)	96.3 (94.9-97.4)	91.3 (88.7-93.4)

Stratum* with lowest predicted proportion (%, 95%CI)	1211	1211	1111	1113	1111	1151	1111	1113	1112
Stratum* with highest predicted proportion (%, 95%CI)	3152	3152	3251	3253	3253	3222	3252	3252	3252
Tetanus vaccination									
VPC (empty model) (%)	7.8	6.5	1.6	1.7	3.9	0.5	5.6	0.6	15.9
VPC (adjusted model for 4E) (%)	0.0	0.0	0.5	0.0	0.0	0.0	0.2	0.0	0.7
PCV (%)	100	100	71.4	100	100	100	96.2	100	96.0
AUC (%)	67	64	59	65	62	58	63	57	74
Lowest predicted proportion (%, 95%CI)	37.4 (29.4-46.2)	36.6 (31.3-42.1)	55.3 (47.5-63.1)	76.6 (68.7-82.7)	61.6 (56.1-66.7)	82.6 (76.3-87.1)	30.1 (24.6-36.5)	47.3 (41.7-53.1)	19.7 (15.9-23.8)
Highest predicted proportion (%, 95%CI)	83.7 (79.3-87.4)	80.0 (75.2-84.0)	74.3 (68.9-79.1)	90.1 (83.9-93.8)	83.8 (80.9-86.3)	92.6 (90.6-94.1)	68.2 (62.6-74.0)	70.1 (65.4-74.6)	85.6 (80.6-89.5)
Stratum* with lowest predicted proportion (%, 95%CI) *	1113	1111	1111	1113	1221	1133	1111	1123	1112
Stratum* with highest predicted proportion (%, 95%CI) *	3251	3252	3252	3241	3243	3222	3252	2241	3251
Skilled birth attendant									
VPC (empty model) (%)	11.6	19.4	33.0	5.2	24.5	4.1	26.7	26.4	30.6
VPC (adjusted model for 4E) (%)	0.5	0.0	0.4	1.4	0.0	0.0	0.0	0.0	1.0
PCV (%)	95.7	100	99.2	73.2	100	100	100	100	97.8
AUC (%)	72	69	85	67	77	62	73	75	84
Lowest predicted proportion (%, 95%CI)	68.9 (59.0-76.9)	36.3 (31.5-40.8)	40.5 (33.8-48.2)	65.2 (50.2-78.0)	18.3 (15.8-21.2)	84.0 (78.5-88.3)	35.6 (31.0-40.7)	46.1 (40.7-52.6)	5.9 (3.9-8.5)
Highest predicted proportion (%, 95%CI) *	96.8 (94.2-98.3)	92.6 (89.7-94.9)	98.4 (97.2-99.1)	92.6 (86.4-96.0)	88.6 (85.4-91.1)	97.0 (95.9-97.8)	97.5 (96.1-98.4)	98.9 (98.2-99.3)	85.8 (82.2-88.9)
Stratum* with lowest predicted proportion (%, 95%CI) *	1213	1213	1111	2211	1212	1233	1111	1113	1113

Stratum* with highest predicted proportion (%, 95%CI)	3152	3152	3152	3253	3153	3152	3253	3252	3251
Vitamin A supplementation									
VPC (empty model) (%)	3.5	4.3	7.8	2.5	2.9	0.3	5.1	1.4	10.1
VPC (adjusted model for 4E) (%)	0.0	0.0	1.0	0.4	0.0	0.0	0.5	0.0	0.4
PCV (%)	100	100	88.4	82.5	100	100	91.2	100	96.6
AUC (%)	64	61	69	63	61	56	62	59	69
Lowest predicted proportion (%, 95%CI)	69.5 (57.0-79.3)	26.1 (21.4-31.3)	41.1 (32.9-49.8)	36.2 (27.6-45.5)	21.4 (17.7-25.8)	59.5 (55.4-63.8)	46.9 (38.9-55.0)	58.6 (54.0-63.1)	23.1 (18.2-28.5)
Highest predicted proportion (%, 95%CI)	91.7 (88.4-94.0)	60.9 (55.1-66.6)	85.5 (78.9-89.9)	64.3 (54.3-74.2)	55.4 (49.9-60.6)	73.8 (71.0-76.4)	81.4 (73.9-87.4)	79.5 (75.4-83.2)	72.4 (68.3-76.1)
Stratum* with lowest predicted proportion (%, 95%CI)	1143	1131	1111	1132	1112	1112	1111	1121	1123
Stratum* with highest predicted proportion (%, 95%CI)	3231	3252	3243	2251	3241	2251	3253	2153	3251
BCG vaccination									
VPC (empty model) (%)	6.3	7.5	36.4	2.5	2.8	1.3	5.6	2.5	11.3
VPC (adjusted model for 4E) (%)	0.0	0.0	0.0	0.7	0.0	0.1	0.2	0.5	1.5
PCV (%)	100	100	100	71.0	100	89.3	95.9	81.3	87.8
AUC (%)	70	64	85	63	60	57	63	60	70
Lowest predicted proportion (%, 95%CI)	85.4 (76.7-91.5)	28.2 (23.7-32.7)	69.9 (63.9-74.8)	45.9 (36.2-55.1)	28.9 (23.8-34.3)	46.2 (39.7-53.2)	33.7 (28.2-40.2)	51.7 (42.9-60.9)	16.5 (12.7-21.2)
Highest predicted proportion (%, 95%CI)	97.3 (95.5-98.5)	74.9 (60.2-80.0)	99.4 (99.0-99.7)	67.7 (56.1-77.0)	61.9 (55.7-67.6)	69.1 (64.9-72.9)	78.5 (70.9-84.2)	73.2 (66.7-79.3)	71.9 (63.6-78.6)
Stratum* with lowest predicted proportion (%, 95%CI)	1113	1211	1111	1232	1251	1253	1111	1253	1112
Stratum* with highest predicted proportion (%, 95%CI)	3242	3253	2242	2113	3113	2111	3243	3122	3152
DPT3 vaccination									

VPC (empty model) (%)	9.7	8.1	11.5	1.7	3.1	1.0	6.2	1.6	13.6
VPC (adjusted model for 4E) (%)	3.3	0.0	0.0	0.1	0.0	0.2	0.5	0.6	1.1
PCV (%)	68.4	100	100	92.2	100	76.6	91.7	62.1	93.0
AUC (%)	69	66	73	62	60	57	64	59	72
Lowest predicted proportion (%, 95%CI)	67.7 (52.2-80.6)	9.6 (7.3-12.7)	63.0 (58.2-67.8)	34.5 (29.4-40.3)	23.1 (17.8-28.9)	43.32(36.0-50.3)	23.7 (18.0-30.3)	42.1 (34.3-50.4)	9.4 (7.0-12.6)
Highest predicted proportion (%, 95%CI)	93.6 (87.1-97.0)	54.0 (46.7-61.5)	94.3 (92.4-95.7)	55.4 (45.4-65.2)	57.3 (52.3-62.2)	64.9 (59.8-69.8)	67.3 (59.5-74.1)	58.2 (50.9-65.9)	57.6 (51.4-63.4)
Stratum* with lowest predicted proportion (%, 95%CI) *	1111	1111	1111	1212	1151	1253	1111	1131	1112
Stratum* with highest predicted proportion (%, 95%CI) *	3233	3243	2232	2131	3213	2111	3252	2141	3251
MSL vaccination									
VPC (empty model) (%)	6.6	7.2	17.8	1.2	2.9	0.9	4.5	2.7	7.0
VPC (adjusted model for 4E) (%)	0.0	0.2	0.0	0.3	0.0	0.1	0.5	0.2	0.5
PCV (%)	100	98.0	100	73.0	100	87.4	89.3	94.4	93.5
AUC (%)	68	65	78	61	60	57	62	61	66
Lowest predicted proportion (%, 95%CI)	72.3 (63.0-80.5)	13.0 (9.6-17.3)	57.3 (51.8-62.5)	38.0 (30.9-45.9)	24.3 (20.5-28.7)	43.6 (36.8-50.63	27.6 (21.4-34.8)	43.6 (37.4-50.0)	16.4 (13.3-20.1)
Highest predicted proportion (%, 95%CI)	94.3 (91.0-96.5)	52.3 (43.3-60.9)	95.9 (94.4-97.1)	59.5 (48.2-69.4)	55.9 (51.3-60.0)	65.1 (60.4-69.3)	68.0 (58.6-76.0)	65.7 (58.9-71.9)	57.6 (52.2-62.5)
Stratum* with lowest predicted proportion (%, 95%CI)	1113	1111	1111	1232	1221	1253	1111	1211	1112
Stratum* with highest predicted proportion (%, 95%CI)	3251	3243	3242	3121	3233	2112	3243	3143	3251
Access to improved water source									
VPC (empty model) (%)	19.6	13.8	10.7	24.5	23.9	9.3	20.6	24.4	16.7
VPC (adjusted model for 4E) (%)	0.0	0.0	0.0	0.9	0.3	0.0	0.0	0.8	0.5
PCV (%)	100	100	100	97.1	99.0	100	100	97.6	97.2

AUC (%)	76	68	69	78	76	65	74	76	75
Lowest predicted proportion (%, 95%CI)	54.0 (48.3-59.3)	56.8 (50.4-63.2)	55.9 (51.5-60.8)	44.4 (35.5-54.5)	12.9 (10.0-16.4)	72.4 (65.9-77.7)	42.6 (38.7-46.5)	56.0 (48.3-63.5)	32.0 (27.1-37.6)
Highest predicted proportion (%, 95%CI)	98.3 (96.7-99.2)	97.4 (95.6-98.3)	92.0 (89.4-93.9)	97.9 (95.8-99.0)	85.4 (81.0-89.0)	96.8 (95.7-97.6)	97.3 (96.1-98.2)	98.9 (98.2-99.4)	93.5 (91.1-95.2)
Stratum* with lowest predicted proportion (%, 95%CI) *	1211	1113	1212	1213	1212	1213	1212	1112	1112
Stratum* with highest predicted proportion (%, 95%CI) *	3151	3252	3253	3152	3152	3152	3151	3152	3151

	Rwanda	South Africa	Sierra Leone	Tanzania	Uganda	Zambia	Zimbabwe
**ANC1**							
VPC (empty model) (%)	24.8	-	3.3	15.0	0.0	9.6	3.0
VPC (adjusted model for 4E) (%)	0.0	-	0.0	0.0	0.0	3.2	0.0
PCV (%)	100	-	100	100	0.0	69.4	100
AUC (%)	82	50	63	73	64	74	68
Lowest predicted proportion (%, 95%CI)	92.8 (87.8-96.1)	83.3 (67.5-92.4)	68.6 (60.3-75.7)	64.0 (56.3-71.6)	95.6 (91.7-97.7)	86.3 (73.9-93.4)	88.0 (80.9-92.7)
Highest predicted proportion (%, 95%CI)	99.7 (98.6-99.9)	96.1 (84.8-99.1)	90.3 (86.9-93.0)	97.1 (95.6-98.1)	98.8 (97.6-99.5)	98.7 (97.0-99.5)	97.5 (95.7-98.5)
Stratum* with lowest predicted proportion (%, 95%CI)	1213	2121	3153	1111	1241	1111	2211
Stratum* with highest predicted proportion (%, 95%CI)	3141	3213	2242	3242	3152	3233	3152
Tetanus vaccination							
VPC (empty model) (%)	0.3	1.0	-	4.8	2.3	0.7	0.6
VPC (adjusted model for 4E) (%)	0.0	0.0	-	0.0	0.0	0.0	0.0
PCV (%)	100	100	-	100	100	100	100
AUC (%)	57	60	50	67	60	60	58

Lowest predicted proportion (%, 95%CI)	69.3 (62.7-75.3)	27.3 (14.3-46.4)	77.9 (73.9-81.8)	73.9 (66.8-80.3)	64.0 (58.7-69.3)	60.6 (63.3-67.1)	45.3 (27.6-64.5)
Highest predicted proportion (%, 95%CI)	78.8 (73.1-83.4)	77.1 (55.9-89.4)	88.1 (83.4-92.1)	94.0 (91.0-95.9)	87.4 (84.3-89.9)	82.5 (78.4-86.1)	62.1 (56.6-67.4)
Stratum*with lowest predicted proportion (%, 95%CI) *	3112	2253	1241	1111	2131	1111	1212
Stratum* with highest predicted proportion (%, 95%CI) *	1243	1122	2153	3253	3223	3233	3133
Skilled birth attendant							
VPC (empty model) (%)	26.3	17.9	3.9	13.7	12.8	8.8	11.4
VPC (adjusted model for 4E) (%)	0.0	0.0	0.0	0.0	0.03	0.0	0.4
PCV (%)	100	100	100	100	99.8	100	97.1
AUC (%)	77	78	63	73	67	68	70
Lowest predicted proportion (%, 95%CI)	72.2 (65.4-77.5)	79.0 (55.0-91.0)	80.2 (77.2-83.2)	57.8 (51.0-64.1)	61.0 (55.1-66.5)	61.5 (54.4-67.9)	41.5 (24.4-63.2)
Highest predicted proportion (%, 95%CI)	99.6 (99.0-99.8)	98.8 (93.3-99.8)	95.7 (93.5-97.1)	95.7 (93.3-97.2)	94.5 (92.6-95.9)	93.3 (91.2-94.9)	91.8 (87.9-94.5)
Stratum*with lowest predicted proportion (%, 95%CI) *	1212	2111	1212	1112	1211	1213	1111
Stratum* with highest predicted proportion (%, 95%CI) *	3153	3233	3131	3151	3153	3252	3253
Vitamin A supplementation							
VPC (empty model) (%)	1.6	1.1	1.7	2.2	0.7	0.9	0.6
VPC (adjusted model for 4E) (%)	0.0	0.0	0.0	0.0	0.0	0.0	0.0
PCV (%)	100	100	100	100	100	100	100
AUC (%)	62	62	59	62	57	60	59
Lowest predicted proportion (%, 95%CI)	77.9 (70.8-83.7)	60.1 (45.8-73.6)	58.2 (49.7-65.9)	37.5 (30.9-44.1)	58.2 (53.1-63.2)	67.0 (60.6-72.6)	55.6 (34.7-72.7)
Highest predicted proportion (%, 95%CI)	93.6 (90.0-96.0)	86.9 (74.0-93.9)	80.4 (76.1-83.9)	73.7 (68.4-78.2)	70.9 (66.6-75.2)	85.3 (81.4-88.5)	78.4 (73.3-82.8)

Stratum* with lowest predicted proportion (%, 95%CI) *	3112	2152	2123	1113	2113	1122	1111
Stratum* with highest predicted proportion (%, 95%CI) *	1253	3123	3251	3251	1242	3251	3153
BCG vaccination							
VPC (empty model) (%)	-	1.6	0.1	6.7	1.0	1.7	0.6
VPC (adjusted model for 4E) (%)	-	0.0	0.0	0.0	0.0	0.2	0.0
PCV (%)	-	100	100	100	100	90.9	100
AUC (%)	50	61	56	71	58	60	58
Lowest predicted proportion (%, 95%CI)	50.2 (42.9-57.3)	22.9 (10.8-43.3)	56.9 (52.5-61.4)	80.8 (73.3-87.0)	50.8 (45.0-57.3)	48.8 (40.8-57.7)	45.0 (37.4-53.0)
Highest predicted proportion (%, 95%CI)	74.3 (67.5-80.0)	57.4 (46.2-68.8)	74.3 (68.3-79.5)	96.7 (94.1-98.0)	77.2 (73.6-80.5)	74.3 (68.6-79.4)	64.6 (60.0-69.0)
Stratum* with lowest predicted proportion (%, 95%CI) *	1253	1232	1231	1113	1253	1253	2251
Stratum* with highest predicted proportion (%, 95%CI) *	3111	3141	2112	3242	3212	2111	3112
DPT3 vaccination							
VPC (empty model) (%)	0.0	0.9	0.1	11.3	0.3	0.7	0.3
VPC (adjusted model for 4E) (%)	0.0	0.0	0.0	0.0	0.0	0.0	0.0
PCV (%)	88.2	100	100	100	100	100	100
AUC (%)	56	60	56	73	56	58	57
Lowest predicted proportion (%, 95%CI)	50.3 (43.1-57.8)	17.3 (8.1-31.6)	46.7 (42.2-51.6)	69.6 (60.9-77.3)	44.4 (39.2-50.0)	46.5 (39.5-53.7)	34.5 (19.8-54.5)
Highest predicted proportion (%, 95%CI)	73.6 (67.3-79.0)	42.4 (34.6-50.2)	60.7 (54.2-67.1)	96.3 (94.0-97.7)	64.7 (60.3-68.5)	69.3 (63.8-73.9)	60.1 (55.2-64.7)
Stratum* with lowest predicted proportion (%, 95%CI) *	1253	2253	1231	1113	1241	1253	1231

Stratum* with highest predicted proportion (%, 95%CI) *	3111	3122	2122	3251	3212	3111	3112
MSL vaccination							
VPC (empty model) (%)	0.0	1.8	-	5.4	0.3	0.8	0.5
VPC (adjusted model for 4E) (%)	0.0	0.0	-	0.0	0.0	0.0	0.0
PCV (%)	100	100	-	100	100	100	100
AUC (%)	56	61	50	68	56	59	58
Lowest predicted proportion (%, 95%CI)	50.7 (43.5-57.5)	20.2 (8.7-41.0)	47.6 (42.3-52.9)	70.1 (62.5-77.1)	46.9 (40.5-53.2)	44.3 (37.2-51.6)	42.0 (35.0-49.0)
Highest predicted proportion (%, 95%CI)	73.8 (67.6-79.6)	54.7 (42.9-65.4)	60.0 (54.2-65.5)	94.5 (91.7-96.4)	68.3 (62.9-72.8)	69.3 (64.5-73.9)	60.4 (55.9-65.2)
Stratum*with lowest predicted proportion (%, 95%CI) *	1253	1232	1223	1142	1151	1253	2251
Stratum* with highest predicted proportion (%, 95%CI) *	3111	3141	3152	3221	3112	2111	3112
Access to improved water source							
VPC (empty model) (%)	11.0	37.8	13.9	21.3	3.3	15.8	12.2
VPC (adjusted model for 4E) (%)	0.0	0.0	0.0	0.0	0.0	0.5	0.0
PCV (%)	100	100	100	100	100	97.4	100
AUC (%)	67	85	70	75	61	72	71
Lowest predicted proportion (%, 95%CI)	63.8 (57.9-69.2)	0.0*	33.9 (29.0-39.2)	36.2 (30.5-42.3)	64.1 (57.7-69.6)	36.5 (28.1-45.9)	53.2 (46.7-59.5)
Highest predicted proportion (%, 95%CI)	96.1 (93.9-97.5)	99.5 (96.6-99.9)	92.0 (89.5-94.0)	95.3 (93.4-96.8)	90.0 (87.1-92.4)	92.1 (88.9-94.3)	93.3 (90.6-95.2)
Stratum*with lowest predicted proportion (%, 95%CI) *	1212	2253	1213	1213	1241	1213	2113
Stratum* with highest predicted proportion (%, 95%CI) *	3151	3142	3152	3251	3112	3152	3152

%– proportion, CI – confidence interval.

*The four dimensions (4Es) of the strata are presented in the following order: educational level: 1=no education, 2=primary, 3=secondary/higher; employment: 1=not currently employed, 2= currently employed; economic status: 1= poorest, 2=poorer, 3=middle, 4=richer, 5=richest; and empowerment: 1=low, 2=middle, 3=high.

9. **Table S9.** Estimates from multilevel models of co-coverage of at least six interventions among women and children in sub-Saharan Africa 2015-2022 with observations clustered by 4Es and country-level (UHC index) social strata.

	Model 1: empty model OR (95%CI)	Model 2a††: effect of education OR (95%CI)	Model 2b††: effect of employment OR (95%CI)	Model 2c††: effect of economic status OR (95%CI)	Model 2d††: effect of empowerment OR (95%CI)	Model 2e††: effect of country's UHC index OR (95%CI)	Model 2f**: Main effects model OR (95%CI)	Model 3: Model adjusted for covariates¶ OR (95%CI)
**Regression coefficients**								
intercept	1.19 (1.11-1.27)	0.80 (0.73-0.87)	1.07 (0.87-1.32)	0.95 (0.82-1.08)	1.21 (1.08-1.36)	0.93 (0.86-1.01)	0.47 (0.41-0.54)	0.75 (0.65-0.87)
**Educational level**							Ref	Ref
None		Ref					1.64 (1.52-1.76)	1.50 (1.40-1.62)
Primary		1.65 (1.45-1.86)					1.94 (1.80-2.10)	1.53 (1.42-1.66)
Secondary/higher		1.99 (1.76-2.26)						
**Currently employed**								
No			Ref				Ref	Ref
Yes			1.07 (0.94-1.23)				1.08 (1.01-1.15)	1.13 (1.07-1.20)
**Economic status**				Ref				
Poorest				1.18 (0.97-1.44)			Ref	Ref
Poorer				1.28 (1.06-1.56)			1.17 (1.06-1.29)	1.15 (1.04-1.26)
Middle				1.43 (1.17-1.74)			1.26 (1.15-1.39)	1.24 (1.13-1.37)
Richer				1.48 (1.21-1.80)			1.41 (1.28-1.55)	1.40 (1.27-1.54)
Richest							1.45 (1.31-1.60)	1.45 (1.32-1.61)
**Women empowerment**								
Low					Ref		Ref	Ref
Middle					0.99 (0.85-1.17)		0.99 (0.92-1.06)	1.00 (0.93-1.07)
High					0.90 (0.77-1.06)		0.90 (0.83-0.97)	0.93 (0.86-1.01)
**Country with high UHC index**								
No						Ref	Ref	Ref
Yes						1.64 (1.46-1.83)	1.61 (1.52-1.72)	1.61 (1.52-1.72)
**Random effects**								
Strata	103,388	103,388	103,388	103,388	103,388	103,388	103,388	103,388
VPC (%)	5.6	3.2	5.5	5.1	5.5	3.9	1.0	0.9
PCV (%)	-	44.8	0.7	9.9	1.0	30.7	83.6	84.2
AUC (%)	63.1	-	-	-	-	-	63.1	-

†† *Models 2a, 2b, 2c, 2d, 2e were partially adjusted for each individual 4Es variables. **Model 2f was adjusted for all 4Es variables and UHC variable.*

¶ *covariates of adjustment were: age, parity, area of residence, and access to mass media.*

10. Table S10. Five highest and lowest ranked strata (4Es + UHC strata) for predicted proportion of women-children pairs participating in at least six interventions in sub-Saharan Africa, 2015-2022

Rank	stratum	Educational level	Employment	Economic status	Empowerment level	Country with high UHC index	n	Predicted proportion (95%CI)	Observed proportion
Five lowest									
1	11131	None	No	Poorest	High	No	473	17.5 (14.6-21.1)	12.5 (9.5-15.4)
2	11121	None	No	Poorest	Middle	No	1749	23.5 (21.1-26.3)	22.7 (20.7-24.7)
3	11111	None	No	Poorest	Low	No	1076	24.6 (21.8-27.8)	23.3 (20.8-25.8)
4	11221	None	No	Poorer	Middle	No	1141	28.6 (25.4-31.9)	27.5 (24.9-30.1)
5	11211	None	No	Poorer	Low	No	762	29.2 (25.3-32.4)	27.2 (24.0-30.3)
Five highest									
1	32412	Secondary/higher	Yes	Richer	Low	Yes	463	73.4 (69.2-77.2)	74.3 (70.3-78.3)
2	31512	Secondary/higher	No	Richest	Low	Yes	267	72.0 (66.7-76.4)	73.0 (67.7-78.4)
3	31412	Secondary/higher	No	Richer	Low	Yes	241	71.8 (66.9-76.9)	73.4 (67.9-79.0)
4	32312	Secondary/higher	Yes	Middle	Low	Yes	412	70.1 (65.5-73.9)	70.6 (66.2-75.0)
5	32422	Secondary/higher	Yes	Richer	middle	Yes	1339	68.5 (65.2-71.7)	68.3 (65.8-70.7)

CI – confidence interval

11. Table S11. Multilevel models of co-coverage with at least six interventions among women and children in sub-Saharan Africa 2015-2022 adjusted for country (Sensitivity analyses).

	Observations clustered by 4Es-social strata		Observations clustered by 4Es and country-level (UHC index) social strata	
	Model 2e†: Main effects model (adjusted for country) OR (95%CI)	Model 3§: Model adjusted for covariates and country OR (95%CI)	Model 2f‡: Main effects model (adjusted for country) OR (95%CI)	Model 3§: Model adjusted for covariates¶ and country OR (95%CI)
**Regression coefficients**				
intercept	0.15 (0.13 – 0.16)	0.27 (0.23 – 0.31)	0.14 (0.12 – 0.16)	0.24 (0.20 – 0.28)
**Educational level**			Ref	Ref
None	Ref	Ref	1.87 (1.73 – 2.01)	1.66 (1.54 – 1.79)
Primary	1.92 (1.77 – 2.09)	1.71 (1.58 – 1.85)	2.78 (2.58 – 3.01)	2.09 (1 1.93 – 2.26)
Secondary/higher	2.92 (2.69 – 3.18)	2.19 (2.02 – 2.39)		
**Currently employed**				
No	Ref	Ref	Ref	Ref
Yes	1.06 (0.99 – 1.13)	1.12 (1.05 – 1.19)	1.06 (0.99 – 1.12)	1.12 (1.05 – 1.19)
**Economic status**				
Poorest	Ref	Ref	Ref	Ref
Poorer	1.23 (1.11 – 1.36)	1.23 (1.11 – 1.36)	1.25 (1.14 – 1.38)	1.26 (1.14 – 1.38)
Middle	1.31 (1.18 – 1.45)	1.34 (1.21 – 1.48)	1.34 (1.22 – 1.47)	1.36 (1.24 – 1.50)
Richer	1.46 (1.32 – 1.62)	1.54 (1.39 – 1.71)	1.49 (1.36 – 1.64)	1.57 (1.43 – 1.73)
Richest	1.50 (1.35 – 1.66)	1.63 (1.47 – 1.81)	1.50 (1.36 – 1.65)	1.63 (1.48 – 1.80)
**Women empowerment**				
Low	Ref	Ref	Ref	Ref
Middle	0.98 (0.90 – 1.07)	1.02 (0.94 – 1.11)	0.98 (0.91 – 1.06)	1.01 (0.94 – 1.10)
High	1.04 (0.96 – 1.13)	1.05 (0.97 – 1.14)	1.04 (0.97 – 1.12)	1.05 (0.98 – 1.13)
**Country with high UHC index**				
No			Ref	Ref
Yes			2.28 (2.03 – 2.57)	2.89 (2.56 – 3.26)
**Random effects**				
Strata	103,388	103,388	103,388	103,388
VPC (%)	0.56	0.5	0.87	0.85
PCV (%)	92.1	92.38	87.32	84.2
AUC (%)	73.01		73.1	-

%– proportion, CI – confidence interval.

†*Model 2e was adjusted for all 4Es and country variables;* ‡*model 2f was adjusted for all 4Es, UHC index, and country variables;* §*Models 3 were model 2e or model 2f (depending on the context) adjusted for covariate* /¶covariates of adjustment were: *age, parity, area of residence, access to mass media, and country variables).*

12. Prevalence of co-coverage 6+ per country

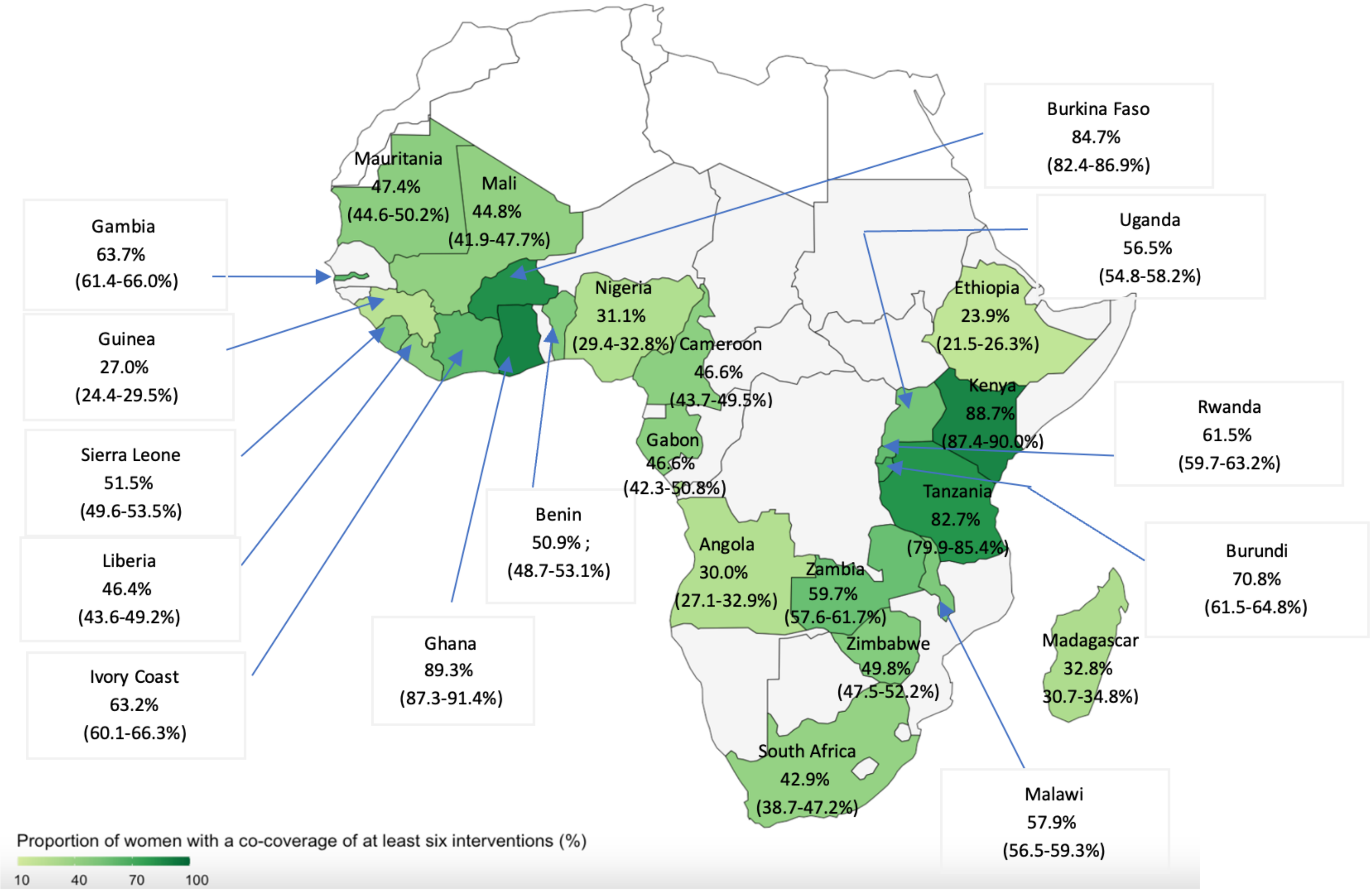

Fig S1. Proportion of women/children pairs with a co-coverage of at least six interventions and its 95% confidence interval per country in sub-Saharan Africa 2015-2022.

13. Analysis of intersectional effects

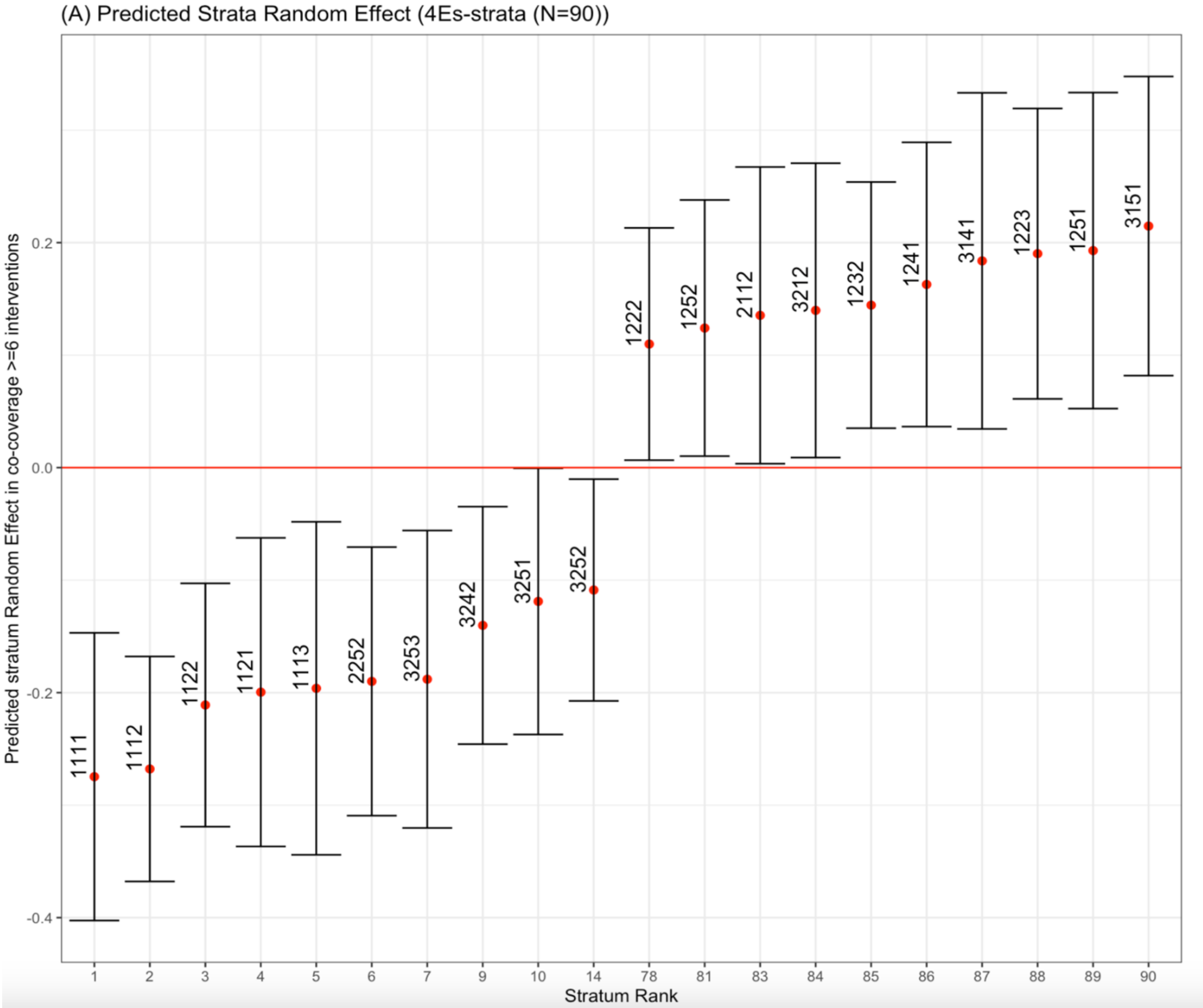

Fig S2. Estimated intersectional effects and their corresponding 95% credible intervals ranked from lowest to highest for co-coverage of six or more interventions. Strata are identified by a four-digit label corresponding to the four dimensions (4Es) in the following order: educational level: 1=no education, 2=primary, 3=secondary/higher; employment: 1=not currently employed, 2= currently employed; economic status: 1= poorest, 2=poorer, 3=middle, 4=richer, 5=richest; and empowerment: 1=low, 2=middle, 3=high.

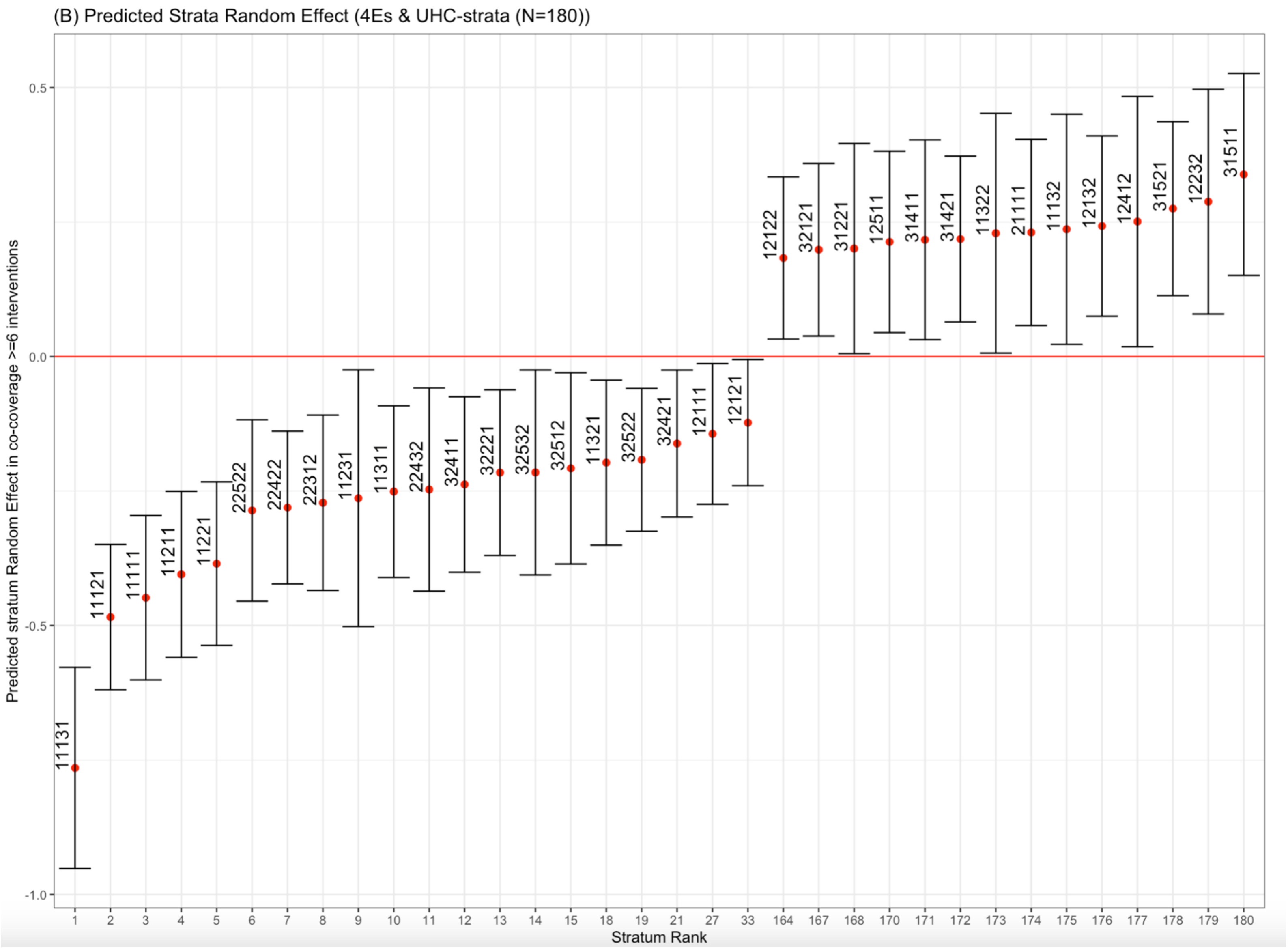

Fig S3. Estimated intersectional effects and their corresponding 95% credible intervals ranked from lowest to highest for co-coverage of six or more interventions. Strata are identified by a four-digit label corresponding to the four dimensions (4Es) in the following order: educational level: 1=no education, 2=primary, 3=secondary/higher; employment: 1=not currently employed, 2= currently employed; economic status: 1= poorest, 2=poorer, 3=middle, 4=richer, 5=richest; and empowerment: 1=low, 2=middle, 3=high; and country with high UHC index: 1= no, 2=yes.

Supplementary material 2: Statistical details

Let y_{ij} denote a binary MNCH outcome (i.e. co-coverage of at least six interventions) for the pair woman/child *i* (*i*=1,…, *n*) in intersectional strata *j* (*j*=1,…., *N*) where:

$$y_{ij} = \begin{matrix} 0 \ absence \ of \ co - coverage \ 6 + \\ 1 \ Presence \ of \ co - coverage \ 6 + \end{matrix}$$

y_{ij} is assumed to follow a Bernoulli distribution, with probabilities $\pi_{ij} = Pr(y_{ij} = 0)$ being the probability of having a co-coverage 6+ (having completed at least six interventions) by the pair woman/child *i* from intersectional stratum *j,* and $1 - \pi_{ij} = Pr(y_i = 1)$, the probability of the pair woman/child *i* of stratum *j* not having completed at least 6 MNCH interventions. . Let X'_{ij} be a vector of explanatory variables used to constitute strata (4Es: Education, Employment, Economic status, Empowerment). The multilevel models are written as follow:

Model 1: with no main effects (the empty model)

As this model is an empty model, there are no differences in its expression for analysis done using observations clustered by 4Es-social strata or using observations clustered by 4Es and country-level (UHC index) social strata.

The multilevel logistic regression model for y_{ij} can be written as:

$$logit(\pi_{ij}) = log\left(\frac{\pi_{ij}}{1-\pi_{ij}}\right) = \beta_0 + \mu_j; \mu_j \sim N(0, \sigma_u^2$$

where β_0 is the log-odds of completing at least six interventions (co-coverage 6+) for an "average" stratum; and μ_j is the magnitude by which social stratum j's log-odds of co-coverage 6+ differs from that of the "average" stratum. This models gives the predicted prevalence of co-coverage 6+ for each stratum by transforming the stratum-specific predicted log-odds into probabilities. These predicted prevalence estimates allow us to understand the intersectional social patterning and to identify any inequalities. The model includes no covariates, so the stratum random effect captures both the main effects of the variables used to define the strata and their two-way and higher order interactions.

The effect of social strata on co-coverage 6+ is measured by the Variance Partition Coefficient (VPC), which represents the proportion of the total variance in the outcome attributable to strata-level differences. The VPC was calculated based on the following equation:

$$\mathrm{VPC} = \frac{\sigma_u^2}{\sigma_u^2 + \pi^2/3} \times 100\%$$

where σ_u^2 is the variance of the stratum-level residuals and $\pi^2/3$ is the non-estimated within-stratum variance term set to equal the variance of the standard logistic distribution. The VPC serves as a measure of discriminatory accuracy, aiding in evaluating the predictive performance of intersectional stratification. In the

case of our study, a higher VPC value indicates that the strata are more heterogeneous in terms of co-coverage 6+ prevalence and individuals within a given stratum are more similar to one another in terms of co-coverage 6+ likelihood.

Model 2: intersectional interaction model

- **Analysis using observations clustered by 4Es-social strata**

The sub-models 2a-e are built upon the model 1 by including indicators for each individual social position category under consideration (minus the referent) as explanatory main effects covariates. The four models (models 2a, 2b, 2c, and 2d) include each individual 4Es variables to appreciate their individual main effect and the fifth model 2e includes all 4Es simultaneously.

The full model (model 2e) is expressed as:

$$logit(\pi_{ij}) = log\left(\frac{\pi_{ij}}{1-\pi_{ij}}\right) = \beta_0 + \beta_{educ}X_{ij}^{educ} + \beta_{emp}X_{ij}^{emp} + \beta_{eco}X_{ij}^{eco} + \beta_{empwr}X_{ij}^{empwr} + \mu_j;\ \mu_j \sim N(0, \sigma_u^2)$$

Where X_{ij}^{educ}, X_{ij}^{emp}, X_{ij}^{eco}, X_{ij}^{empwr} represent vectors of the educational level, employment, economic status, and empowerment indicators, respectively.

The model 2a includes only education and is expressed as:

$$logit(\pi_{ij}) = log\left(\frac{\pi_{ij}}{1-\pi_{ij}}\right) = \beta_0 + \beta_{educ}X_{ij}^{educ} + \mu_j;\ \mu_j \sim N(0, \sigma_u^2)$$

The model 2b includes only employment and is expressed as:

$$logit(\pi_{ij}) = log\left(\frac{\pi_{ij}}{1-\pi_{ij}}\right) = \beta_0 + \beta_{emp}X_{ij}^{emp} + \mu_j;\ \mu_j \sim N(0, \sigma_u^2)$$

The model 2c includes only economic status and is expressed as:

$$logit(\pi_{ij}) = log\left(\frac{\pi_{ij}}{1-\pi_{ij}}\right) = \beta_0 + \beta_{eco}X_{ij}^{eco} + \mu_j;\ \mu_j \sim N(0, \sigma_u^2)$$

The model 2d includes only empowerment and is expressed as:

$$logit(\pi_{ij}) = log\left(\frac{\pi_{ij}}{1-\pi_{ij}}\right) = \beta_0 + \beta_{empwr}X_{ij}^{empwr} + \mu_j;\ \mu_j \sim N(0, \sigma_u^2)$$

The inclusion of these variables aims at adjusting for the additive effects of each 4Es, to estimate their intersectional interaction effects. In fact, any residual strata-level variance represents two-way or higher interactions between these four dimensions of social position. The model allows to calculate the predicted excess prevalence due to interaction by isolating each stratum's residual term and positive value indicates that

a given stratum has a higher predicted prevalence than expected based on the additive effects, whereas a negative value indicates that the stratum has a lower predicted prevalence than expected. In addition to the VPC, the Proportional Change in Variance (PCV) which quantifies the proportion of the total between-stratum variance (as quantified by the VPC from the model 1) that is attributable to the additive effects, was calculated:

$$\mathrm{PCV} = \frac{\sigma^2_{u(1)} - \sigma^2_{u(2)}}{\sigma^2_{u(1)}} \times 100\%$$

where $\sigma^2_{u(1)}$ and $\sigma^2_{u(2)}$ denote the variance of the stratum-level residuals from the model 1 and model 2a-e respectively. The VPC represents the proportion of the total between-stratum variance of intersectional strata of the empty model that is explained after including main effects. Thus, in the absence of any stratum specific interactions, the main effects used to construct the intersectional strata would completely explain the between-stratum variance and all stratum random effects would be equal to zero. Consequently, a higher PCV indicates that the additive effects explain more of the between-stratum variance and a lower PCV implies that a higher amount of variance is explained either by interaction effects or omitted variable bias. Collectively, the estimates obtained from this model will help in evaluating the extent to which intersectional interactions between the 4Es contribute to the social patterning of the co-coverage 6+, both for specific strata (i.e., the excess prevalence estimates) and at the population-level (i.e., the PCV).

A sensitivity analysis was performed by adding the country variable to the 2e model. It was then expressed as:

$$logit(\pi_{ij}) = log\left(\frac{\pi_{ij}}{1-\pi_{ij}}\right) = \beta_0 + \beta_{educ}X_{ij}^{educ} + \beta_{emp}X_{ij}^{emp} + \beta_{eco}X_{ij}^{eco} + \beta_{empwr}X_{ij}^{empwr} + \beta_{country}X_{ij}^{country} + \mu_j;$$
$$\mu_j \sim N(0, \sigma_u^2)$$

Where $X_{ij}^{country}$ represents the vector of the country variable.

- **Analysis using observations clustered by 4Es and country-level (UHC) social strata**

Like above, the sub-models 2a-f are built upon the model 1 by including indicators for each individual social position category and the UHC index as explanatory main effects covariates. The four models (models 2a, 2b, 2c, and 2d) include each individual 4Es variables and the fifth (model 2e) includes the country's UHC index variable to appreciate their individual main effect. The sixth (model 2f) includes all 4Es + country's UHC index simultaneously.

The models 2a, 2b, 2c, and 2d are expressed like above. The model 2e includes only country's UHC index and is expressed as:

$$logit(\pi_{ij}) = log\left(\frac{\pi_{ij}}{1-\pi_{ij}}\right) = \beta_0 + \beta_{uhcindex}X_{ij}^{uhcindex} + \mu_j;\ \mu_j \sim N(0, \sigma_u^2)$$

Where $X_{ij}^{uhcindex}$ represents the vector of the UHC index.

The full model (model 2f) is then expressed as:

$$logit(\pi_{ij}) = log\left(\frac{\pi_{ij}}{1-\pi_{ij}}\right) = \beta_0 + \beta_{educ}X_{ij}^{educ} + \beta_{emp}X_{ij}^{emp} + \beta_{eco}X_{ij}^{eco} + \beta_{empwr}X_{ij}^{empwr} + \beta_{uhcindex}X_{ij}^{uhcindex} + \mu_j;$$
$$\mu_j \sim N(0, \sigma_u^2)$$

A sensitivity analysis was performed by adding the country variable to the 2f model. It was then expressed as:

$$logit(\pi_{ij}) = log\left(\frac{\pi_{ij}}{1-\pi_{ij}}\right) = \beta_0 + \beta_{educ}X_{ij}^{educ} + \beta_{emp}X_{ij}^{emp} + \beta_{eco}X_{ij}^{eco} + \beta_{empwr}X_{ij}^{empwr} + \beta_{uhcindex}X_{ij}^{uhcindex} +$$
$$\beta_{country}X_{ij}^{country} + \mu_j; \mu_j \sim N(0, \sigma_u^2)$$

Where $X_{ij}^{country}$ represents the vector of the country variable.

Model 3: the adjusted intersectional interaction

Finally, the third model was built upon the model 2e (if observations clustered by 4Es-social strata) or the model 2f (if observations clustered by 4Es and country-level social strata) by including age, parity, area of residence, and access to mass media as explanatory covariates. Two sensitivity analyses were performed by adjusting for the country variable. The same estimates as those obtained from models 2e or 2f, were obtained from this model.
